# Population genomics of the introduced and cultivated Pacific kelp *Undaria pinnatifida*: Marinas—not farms—drive regional connectivity and establishment in natural rocky reefs

**DOI:** 10.1111/eva.12647

**Published:** 2018-06-14

**Authors:** Jaromir Guzinski, Marion Ballenghien, Claire Daguin‐Thiébaut, Laurent Lévêque, Frédérique Viard

**Affiliations:** ^1^ Laboratory Adaptation and Diversity in Marine Environments (UMR 7144 CNRS SU) CNRS Sorbonne Université Roscoff France; ^2^ Laboratory Evolutionary Biology and Ecology of Algae (UMI 3614 CNRS SU) CNRS Sorbonne Université Roscoff France; ^3^ Fédération de Recherche (FR 2424 CNRS SU) CNRS Sorbonne Université Roscoff France

**Keywords:** adaptation, artificial habitats, gene flow, genotyping by sequencing, invasive species, rocky reefs

## Abstract

Ports and farms are well‐known primary introduction hot spots for marine non‐indigenous species (NIS). The extent to which these anthropogenic habitats are sustainable sources of propagules and influence the evolution of NIS in natural habitats was examined in the edible seaweed *Undaria pinnatifida*, native to Asia and introduced to Europe in the 1970s. Following its deliberate introduction 40 years ago along the French coast of the English Channel, this kelp is now found in three contrasting habitat types: farms, marinas and natural rocky reefs. In the light of the continuous spread of this NIS, it is imperative to better understand the processes behind its sustainable establishment in the wild. In addition, developing effective management plans to curtail the spread of *U. pinnatifida* requires determining how the three types of populations interact with one another. In addition to an analysis using microsatellite markers, we developed, for the first time in a kelp, a ddRAD‐sequencing technique to genotype 738 individuals sampled in 11 rocky reefs, 12 marinas, and two farms located along ca. 1,000 km of coastline. As expected, the RAD‐seq panel showed more power than the microsatellite panel for identifying fine‐grained patterns. However, both panels demonstrated habitat‐specific properties of the study populations. In particular, farms displayed very low genetic diversity and no inbreeding conversely to populations in marinas and natural rocky reefs. In addition, strong, but chaotic regional genetic structure, was revealed, consistent with human‐mediated dispersal (e.g., leisure boating). We also uncovered a tight relationship between populations in rocky reefs and those in nearby marinas, but not with nearby farms, suggesting spillover from marinas into the wild. At last, a temporal survey spanning 20 generations showed that wild populations are now self‐sustaining, albeit there was no evidence for local adaptation to any of the three habitats. These findings highlight that limiting the spread of *U. pinnatifida* requires efficient management policies that also target marinas.

## 1 INTRODUCTION

Nonindigenous species (NIS) exert a plethora of effects on native flora and fauna, not the least of which is the breakdown of biogeographical boundaries and biotic homogenization (Capinha, Essl, Seebens, Moser, & Pereira, 2015), trends unlikely to slow down in the near future (Seebens et al., 2017). In marine systems, maritime traffic and aquaculture perpetuate biological introduction processes at global and regional scales (Minchin, 2007a; Molnar, Gamboa, Revenga, & Spalding, 2008; Nunes, Katsanevakis, Zenetos, & Cardoso, 2014; Savini et al., 2010). Marinas form dense networks along the coasts and host diverse and abundant NIS taxa (e.g., sessile NIS in marinas of the Celtic Sea, Bishop, Wood, Lévêque, Yunnie, & Viard, 2015; Minchin, 2007b). As such, they are putatively an important source of propagules for the colonization of neighbouring natural habitats (Bulleri & Chapman, 2010; Glasby, Connell, Holloway, & Hewitt, 2007). Similar spillover effects can occur from farmed NIS, as exemplified in the well‐studied Pacific oyster *Crassostrea gigas* (or *Magallana gigas* according to the World Register of Marine Species, Costello et al., 2013) in the NE Atlantic (Troost, 2010), or the Atlantic salmon *Salmo salar* in the NE Pacific (Fisher, Volpe, & Fisher, 2014; Glover et al., 2017). Our working hypothesis is that the sustainable establishment of NIS in natural habitats relies on spillover and/or recurrent propagule pressure (i.e., a source–sink model) from these anthropogenic habitats.

DNA‐based studies can shed light on the eco‐evolutionary processes sustaining successful introductions and establishment of NIS (Bock et al., 2015; Rius, Turon, Bernard, Volckaert, & Viard, 2015), and guide policies directed towards the prevention or the management of NIS (Darling et al., 2017). They can provide evidence for the ‘spillover’ and ‘source–sink’ processes mentioned above, currently largely investigated with indirect approaches using field survey methods. Few population studies have examined the links, such as connectivity patterns, between marine NIS populations established in artificial (other than farms) and wild habitats (Bishop et al., 2017; Fauvelot, Bertozzi, Costantini, Airoldi, & Abbiati, 2009). Nonetheless, this knowledge is of paramount importance for the development of effective management and mitigation measures and, in particular, to define priority targets. Determining the extent of postintroduction adaptations, and their underlying mechanisms, is also still an important knowledge gap, with little evidence provided thus far in marine systems (Viard, David, & Darling, 2016). Investigating genomic variation in populations living in different habitat types may provide insight into adaptive processes. Furthermore, marinas are known to display specific abiotic features, species assemblages and ecosystem functioning (e.g., Airoldi, Turon, Perkol‐Finkel, & Rius, 2015; Leclerc & Viard, 2018; Megina, González‐Duarte, López‐González, & Piraino, 2013; Ros, Vasquez‐Luis, & Guerra‐Gardia, 2013). Therefore, the selective pressures operating in these artificial habitats are likely very different compared with the nearby natural habitats. Similar to that, cultivated NIS undergo artificial selection, even unintentional, for traits such as increased growth rate or biomass (Valero et al., 2017), which may have substantial impacts on life‐history traits and genetic diversity, as shown in the red alga *Gracillaria chilensis* (Guillemin et al., 2008). However, recurrent propagule pressure from these artificial habitats and/or human‐controlled populations may impede the evolution towards local adaptation in wild habitats.

In this context, the seaweed *Undaria pinnatifida* (Harvey) Suringar, 1873, also known as wakame, is an interesting case. Human‐mediated dispersal has enabled this brown alga to become an extremely successful worldwide invasive species: over the past 40–50 years, this species, native to the NW Pacific, has become well established along the coastlines of North and South America, Australia, New Zealand, and Europe (Epstein & Smale, 2017b; Minchin & Nunn, 2014; South, Floerl, Forrest, & Thomsen, 2017). *U. pinnatifida* is one of 346 seaweed species that have been classified as invasive (Thomsen, Wernberg, South, & Schiel, 2016). Although being a very successful NIS, its impacts on the shallow coastal communities seem often moderate and/or limited to taxa with similar properties both in Australia and Europe (Epstein & Smale, 2017b; South et al., 2017).

Successful invasive seaweeds comprise multiple species that do not appear to share particular properties or traits (Thomsen et al., 2016; Valentine, Magierowski, & Johnson, 2008). However, several life‐history traits have been identified as key to rendering *U. pinnatifida* a successful invader (South et al., 2017; Wallentinus, 2007). It is an opportunistic species which can rapidly colonize disturbed habitats, as shown by experimental removal of native canopies (De Leij, Epstein, Brown, & Smale, 2017; South & Thomsen, 2016; Valentine & Johnson, 2003). This kelp displays a haploid–diploid life cycle, consisting of a large diploid sporophyte phase alternating with a microscopic haploid gametophyte phase. Conversely to other invasive seaweeds with a similar life cycle (e.g., *Gracillaria vermiculophylla*; Krueger‐Hadfield et al., 2016), there is no evidence of vegetative reproduction in introduced populations of *U. pinnatifida* but it is a self‐compatible sexually reproducing species, a trait that may facilitate colonization of new habitats (Pannell et al., 2015). In addition, it displays high fecundity and a short generation time (e.g., two generations per year in Brittany [north western France], Castric‐Fey, Beaupoil, Bouchain, Pradier, & L'Hardy‐Halos, 1999). It can tolerate a wide range of physiological conditions (see references in South et al., 2017) and displays a broad ecological niche (Murphy, Johnson, & Viard, 2017). On the other hand, two characteristics are expected to limit its expansion. First, *U. pinnatifida* is usually less abundant in dense native macroalgal canopies. The native macroalgal canopies seem to resist invasion by *U. pinnatifida*, suggesting that this NIS is poorly competitive (De Leij et al., 2017; South & Thomsen, 2016). Second, spores and gametes of *U. pinnatifida* have very short life duration, thus limiting its ability to disperse by these natural means to distances of 1–100 m, although possible longer distance dispersal (1–10 km) might be possible through drifting mature thalli (Forrest, Brown, Taylor, Hurd, & Hay, 2000; Grulois, Lévêque, & Viard, 2011; Sliwa, Johnson, & Hewitt, 2006).

Most *U. pinnatifida* populations around the world, including in Europe, have been reported in marinas, particularly on floating pontoons, where this alga can reach high densities (e.g., up to 50–60 individuals per m² in Brittany, M. Salomon, L. Lévêque, M. Ballenghien, & F. Viard, unpublished data). A recent field‐based survey carried out in the English Channel showed a relationship between the distance to marinas and presence/abundance of this kelp into the wild (Epstein & Smale, 2017a), supporting a scenario of spillover from marinas to the surrounding natural habitats (i.e., rocky reefs). Along the French coasts of the English Channel, farming activities are additional sources of spread into the wild. This species, accidentally introduced in the Thau Lagoon (Mediterranean Sea) in 1971 (Perez, Lee, & Juge, 1981), was subsequently deliberately introduced in the early 1980s to several locations along the coast of Brittany for cultivation. Soon after these farms were set up, individuals escaped into the wild (Castric‐Fey, Girard, & L'Hardy‐Halos, 1993; Floc'h, Pajot, & Mouret, 1996). A worldwide genetic study suggested that the few *U*. *pinnatifida* farms in Brittany may have been the primary source of many European populations, conversely to other regions where commercial vessels appear to be the main introduction vectors (Epstein & Smale, 2017b; South et al., 2017; Voisin, Engel, & Viard, 2005). In Europe, additional introductions and spread by maritime traffic may have since occurred (Epstein & Smale, 2017b; Fletcher & Farrell, 1999). Today, this kelp can be found along the European Atlantic coast from Portugal to as far north as Belfast Lough, Northern Ireland (Minchin & Nunn, 2014). Its range is predicted to expand further in the British Isles and along the Norwegian Sea coast (Minchin & Nunn, 2014; Murphy et al., 2017).

Some 40 years, that is, 80 generations, after its initial introduction, *U*. *pinnatifida* is well established, particularly in marinas and less frequently in the wild rocky reefs across the entire coastline of Brittany. It is also still farmed in two French bays, where the species has been cultivated for ca. 20–30 years. The present‐day relationship between populations found in marinas, natural rocky reefs and cultivated populations is unclear. In particular, it is uncertain whether the sustainable establishment of the populations found in the wild, most often characterized by low population density (L. Lévêque & F. Viard, unpublished data), still relies on the dense populations found in the nearby marinas or farms. This knowledge is needed to address concerns related to possible intensification of this kelp's cultivation in Europe, as well as to define targets for management strategies to limit its spread (South et al., 2017). On a broader scale, *U*. *pinnatifida* offers an interesting case study on the spillover and adaptive processes in marine NIS.

Considering the short life cycle, the likely limited dispersal distance by spores and gametes, and the high population density of *U. pinnatifida* in marinas and farms, we hypothesized that: (a) human‐mediated dispersal, particularly through leisure boating, plays a prominent role in connectivity patterns at a regional scale (the lack of relationship between the genetic and geographic distances would support this hypothesis); (b) the sustainable establishment of wild populations depends on immigrants from anthropogenic habitats (marinas or farms), the alternative hypothesis being that wild populations are now self‐sustaining; and (c) considering the long time elapsed since the introduction (80 generations in the study range), signs of local adaptation, either contrasting northern and southern populations (Brittany is a transition zone between two biogeographical provinces) or habitats, should be observed, except if counterbalanced by high inter‐habitat or inter‐province gene flow.

These questions can be efficiently addressed using population genomics approaches (Viard et al., 2016), particularly in species that show relatively low polymorphism such as *U*. *pinnatifida* (Daguin, Voisin, Engel, & Viard, 2005; Grulois et al., 2011). In addition, genome‐wide investigation increases the likelihood of identifying outlier loci (i.e., loci that display levels of population differentiation lower or higher than that under neutral expectations). These outliers being under selection, or more likely linked with a locus under selection (Bierne, Welch, Loire, Bonhomme, & David, 2011), are informative regarding local adaptation. We, thus, genotyped 738 *U*. *pinnatifida* sporophytes using a modified double‐digest restriction site‐association DNA (ddRAD) sequencing technique (Brelsford, Dufresnes, & Perrin, 2016). To our knowledge, this is the first population genomics study of this worldwide invader and, more globally, seaweeds. A greater number of individuals collected from the same localities were also examined with a set of 10 microsatellite loci for comparisons with a previous study carried out at the bay scale (Grulois et al., 2011) and the ddRAD‐seq panel. The study individuals were sampled from 25 localities spread across ca. 1,000 km of Brittany coastline and comprising the three different habitat types (natural rocky reefs, marinas, and farms). In addition, we used DNA obtained in 2005 and 2009, 20 and 12 generations earlier respectively, to investigate temporal changes in genetic composition in each of these three habitat types in a single bay.

## 2 MATERIALS AND METHODS

### 2.1 Sampling and DNA extraction

**Figure 1:**
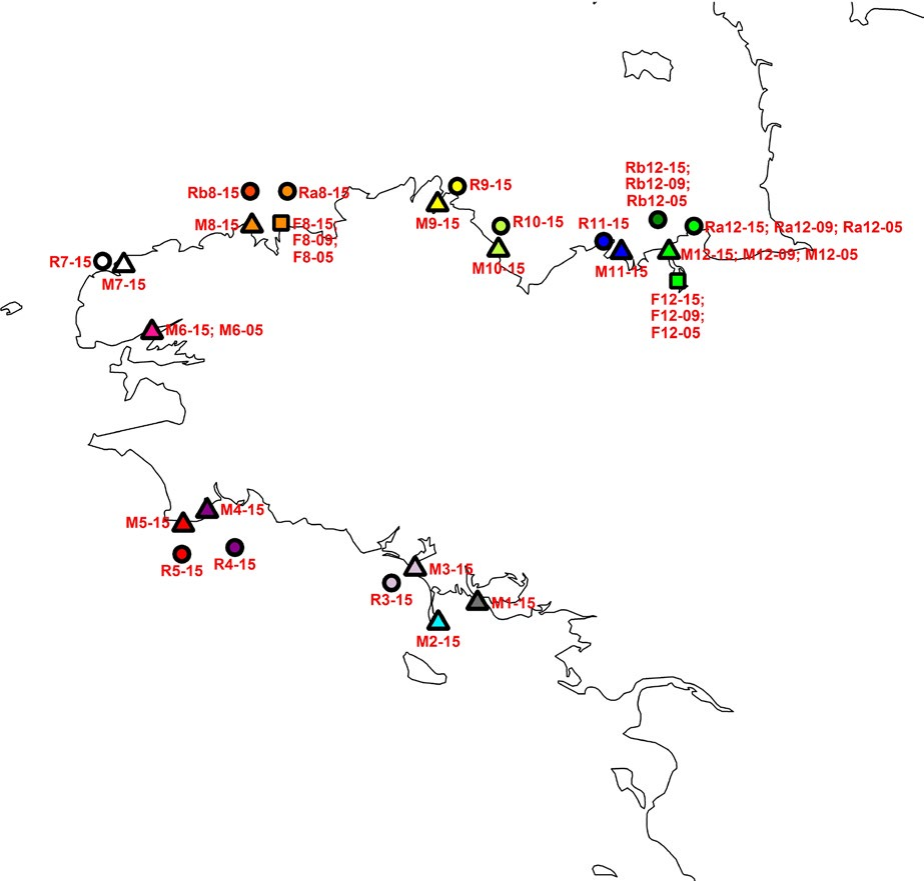
Study area (Brittany, France) and sampling localities for each habitat type. Triangles, circles and squares represent marinas, natural habitats and farms, respectively. Each site's code indicates the habitat type (M, marina; R, natural rocky reef; F, farm), the bay (no. 1–12) and the year of sampling (2005, 2009 and 2015) (e.g., M8‐15 indicates that this site is a marina within bay no. 8 sampled in 2015). The colour code refers to the colours used in the DAPC analysis shown in Supporting Information Figure S2. The geographic name of each locality and bay are detailed in Table 1

From 25 to 148 mature sporophytes were sampled from each of 25 localities, comprising 11 natural rocky reef sites, 12 marinas, and two farms, between January and May 2015, to examine *U*. *pinnatifida* genetic variation across space (Table 1, Figure 1). These 25 localities, distributed across 12 bays, are representative of the present‐day distribution of *U*.* pinnatifida* in Brittany (Araújo et al., 2016; Epstein & Smale, 2017b). The sampling focused on neighbouring (i.e., occurring within the same bay) pairs of natural rocky reef and marina habitats whenever possible. There were seven such pairs in our data set. In some bays, such as the Bay of Brest (bay no. 6 in Figure 1) or around La Trinité‐Sur‐Mer (no. 1 in Figure 1), no wild populations have been reported so far. In Brittany, *U*.* pinnatifida* is currently cultivated in three different bays, but in only two of them has this kelp been cultivated for a long time: since ca. 1980s in the Bay of St. Malo (no. 12 in Figure 1), and since the 1990s‐early 2000s in the Bay of Morlaix (no. 8 in Figure 1). We sampled close‐by trios of populations from marinas, rocky reefs and farms in each of these two bays (Table 1). Total genomic DNA was extracted from up to 32 individuals per sample from approximately 20 mg of silica gel‐dried tissue. Extractions were performed using the Nucleospin^®^ 96 plant kit (Macherey‐Nagel, Düren, Germany) according to the manufacturer's instructions, but with a lysis step at room temperature instead of at 65°C to avoid extracting too many polysaccharides. DNA was eluted in two successive and separate steps with 100 μl elution buffer.

**Table 1:** Sample description with genetic diversity and selfing rate estimates computed on 10,615 SNPs

Site	Habitat	Locality (Bay)	*N*_ind_	*H*_E_ (*SE*)	*F*_IS_	*p*_HW_	*s*
M1‐15	Marina	Port‐Navalo (La Trinité‐Sur‐Mer)	22	0.118 (0.002)	0.334	<0.001	0.436
M2‐15	Marina	Port Haliguen (Quiberon)	17	0.118 (0.002)	0.465	<0.001	0.662
M3‐15	Marina	Port d'Etel (Etel)	19	0.126 (0.002)	0.225	<0.001	0.297
R3‐15	Rocky reef	Magouër Nord (Etel)	20	0.131 (0.002)	0.147	<0.001	0.203
M4‐15	Marina	Port de Loctudy (Loctudy)	15	0.076 (0.002)	0.405	<0.001	0.546
R4‐15	Rocky reef	Karreg Saoz (Loctudy)	23	0.079 (0.002)	0.022	<0.001	0.117
M5‐15	Marina	Port Le Guilvinec (Le Guilvinec)	23	0.107 (0.002)	0.227	<0.001	0.400
R5‐15	Rocky reef	Le Guilvinec Château (Le Guilvinec)	20	0.100 (0.002)	0.112	<0.001	0.249
M6‐15	Marina	Moulin Blanc (Brest)	19	0.120 (0.002)	0.371	<0.001	0.505
M6‐05	Marina	Moulin Blanc (Brest)	19	0.114 (0.002)	0.232	<0.001	0.356
M7‐15	Marina	Port Aber Wrac'h (Aber Wrac'h)	20	0.112 (0.002)	0.182	<0.001	0.291
R7‐15	Rocky reef	Breach ver (Aber Wrac'h)	23	0.110 (0.002)	0.119	<0.001	0.198
M8‐15	Marina	Port Bloscon (Morlaix)	24	0.139 (0.002)	0.125	<0.001	0.243
F8‐15	Farm	Ferme Biocean (Morlaix)	24	0.068 (0.002)	−0.129	<0.001	0.188
F8‐09	Farm	Ferme Biocean (Morlaix)	21	0.113 (0.002)	0.016	1.000	0.278
F8‐05	Farm	Ferme Biocean (Morlaix)	19	0.087 (0.002)	−0.033	0.868	0.255
Ra8‐15	Rocky reef	Guerhéon (Morlaix)	24	0.111 (0.002)	0.158	<0.001	0.355
Rb8‐15	Rocky substrate	Men Guen (Morlaix)	23	0.037 (0.001)	0.090	<0.001	0.238
M9‐15	Marina	Port Trieux (Bréhat)	20	0.129 (0.002)	0.260	<0.001	0.466
R9‐15	Rocky reef	Chenal Ile (Bréhat)	16	0.097 (0.002)	0.101	<0.001	0.153
M10‐15	Marina	Port St. Quay (St. Quay Portrieux)	21	0.055 (0.001)	0.279	<0.001	0.323
R10‐15	Rocky reef	Ile Harbour (St. Quay Portrieux)	21	0.079 (0.002)	0.283	<0.001	0.541
M11‐15	Marina	Port St. Cast (St. Cast le Guildo)	2	0.164 (0.003)	0.438	1.000	0.052
R11‐15	Rocky reef	Roche de l'Etendrée (Frehel)	23	0.125 (0.002)	0.101	<0.001	0.219
M12‐15	Marina	Port Bas Sablons (St. Malo)	23	0.151 (0.002)	0.282	<0.001	0.308
M12‐09	Marina	Port Bas Sablons (St. Malo)	20	0.137 (0.002)	0.216	<0.001	0.369
M12‐05	Marina	Port Bas Sablons (St. Malo)	20	0.124 (0.002)	0.276	<0.001	0.355
F12‐15	Farm	C‐weed (St. Malo)	24	0.067 (0.002)	−0.072	<0.001	0.261
F12‐09	Farm	C‐weed (St. Malo)	22	0.102 (0.002)	0.070	<0.001	0.296
F12‐05	Farm	C‐weed (St. Malo)	21	0.119 (0.002)	‐0.008	1.000	0.284
Ra12‐15	Rocky reef	Fort National (St. Malo)	22	0.140 (0.002)	0.096	<0.001	0.157
Ra12‐09	Rocky reef	Fort National (St. Malo)	22	0.139 (0.002)	0.040	<0.001	0.128
Ra12‐05	Rocky reef	Fort National (St. Malo)	23	0.135 (0.002)	0.267	<0.001	0.420
Rb12‐15	Rocky reef	Le Grand Murier (St. Malo)	22	0.146 (0.002)	0.094	0.002	0.251
Rb12‐09	Rocky reef	Le Grand Murier (St. Malo)	19	0.137 (0.002)	0.121	0.150	0.291
Rb12‐05	Rocky reef	Le Grand Murier (St. Malo)	19	0.145 (0.002)	0.170	<0.001	0.279

*Note*

The locality label is composed of the habitat type (M = marina, R = natural rocky reef, F = cultivated population), the bay number code, and the year of sampling as shown in Figure 1. For each locality, the number of study individuals, the expected heterozygosity (*H*_E_), the estimate of fixation index *F*_IS_ and the probability of the exact test for Hardy–Weinberg equilibrium (pHW) are provided. Selfing rates (*s*) estimated with the *g*_2_ value (David et al., 2007) are also given. Similar information is provided in the Supplementary Material (Supporting Information Table S1) for the microsatellite panel.

In addition, DNA obtained in previous studies (Grulois et al., 2011; Voisin et al., 2005) was used to examine temporal changes. We included individuals originating from five localities (all four localities from the Bay of St. Malo [no. 12], and the farm from the Bay of Morlaix [no. 8]) that were collected in 2005 and in 2009, and from one locality (Brest marina, no. 6) that was sampled in 2005, thus adding a total of 11 temporal samples to our study (Table 1). To increase the number of the RAD library samples for two localities (F8‐05 and M12‐05; Table 1), we included DNA from six and five individuals collected in 2006, respectively.

### 2.2 RAD library construction and SNP calling

Double‐digest RAD‐seq libraries were constructed with *Pst*I and *Mse*I according to the protocol detailed in Brelsford et al. (2016), after fluorometric quantification of DNA concentration with PicoGreen (Invitrogen, Carlsbad, CA, USA) and normalization of the extracts. In total, 738 individuals from 36 populations (spatial or temporal) were sequenced in eight libraries. Each library was sequenced in a single lane of an Illumina HiSeq 2500 v4 flow cell, generating 125‐bp single‐end reads, at Eurofins Genomics (Ebersberg, Germany). Two samples were replicated in each of the eight libraries, and one sample was replicated in six of them, so that a total of 757 samples were sequenced.

In total, sequencing produced 1,743,297,805 reads across the eight libraries. Within each library, the reads were demultiplexed by the index (performed at Eurofins Genomics) and by the barcode with the *process_radtags* programme of Stacks 1.35 (Catchen, Hohenlohe, Bassham, Amores, & Cresko, 2013). Afterwards, we ran cutadapt 1.8.3 (Martin, 2011) to remove the reads containing adaptors and to trim the 5′ *Pst*I overhang nucleotides from the beginning of each read. We used the Stacks *denovo_map.pl* wrapper for locus assembly and Single‐Nucleotide Polymorphism (SNP) calling. Parameters were chosen to limit the risk of assembling non‐homologous loci, a necessary precaution in the absence of a reference genome. We, thus, used a minimal stack depth of five (‐m parameter), up to two mismatches within a locus at the sample level (‐M parameter), up to two mismatches when merging loci across the 757 sample data set (‐n parameter), and up to four mismatches when aligning secondary reads to primary stacks (‐N parameter). At last, using the ‐r filter of the Stacks *populations* programme, the only loci selected were those that occurred in at least 75% of the 757 samples. Quality of SNP calling was assessed by comparing genotypes across replicates, with genotype congruency averaging 99.5%. Further filtering steps were performed in R‐3.3.2. (R Development Core Team 2016) on the variant calling format (VCF) file exported from *populations*. These included the following: (a) keeping only those loci with a maximum of two SNPs per locus; (b) randomly selecting a single SNP per locus; (c) removing three individuals with very low number of loci (missing data exceeding 95%); and (d) discarding loci with global minor allele frequency (MAF) below 0.01 or within‐population MAF below 0.1 using the filter_maf function of the *stackr* R package (Gosselin & Bernatchez, 2016). The final data set comprised 10,615 single‐SNP loci polymorphic in a sample of 735 *U*. *pinnatifida* sporophytes originating from 36 temporal or spatial samples. There was 11.62% missing data across the data set. Note that M11‐15 has only two individuals in the final data set due to failure to amplify the vast majority of samples from this population. Conversion of data from VCF to different input formats was performed in R or in PGDSpider (Lischer & Excoffier, 2011).

### 2.3 Microsatellite genotyping

A total of 1,111 individuals originating from the same 36 temporal or spatial samples genotyped with the RAD‐seq markers were genotyped with 10 microsatellite loci: Up‐AC‐1B2, Up‐AC‐1B5, Up‐AC‐1C1, Up‐AC‐1G2, Up‐AC‐1H5, Up‐AC‐2C1, Und_2E8, Up‐AC‐4G2, Up‐AC‐4C12, Up‐AC‐4E9 (Daguin et al., 2005). Of these 1,111 individuals, 706 were used in the RAD‐seq study. PCR amplification was carried out as detailed in Grulois et al. (2011). Amplification products were separated by electrophoresis on an ABI 3130 XL capillary sequencer (Applied Biosystems, USA). Genotypes were scored using GeneMapper^®^ v. 4.0 (Applied Biosystems). All the analyses described below for the RAD‐seq panel were also performed on the microsatellite panel unless specified otherwise.

### 2.4 Population diversity and mating system statistics

For each of the 36 temporal or spatial samples, the expected heterozygosity, *H*_e_, and the fixation index, *F*_IS_, were estimated in the *genepop* 1.0 (Rousset, 2008) R package. The same software was used to test for departures from Hardy–Weinberg equilibrium in each sample, with the *p*‐values computed using enumeration for the RAD‐seq panel, and using the Markov chain algorithm (100,000 dememorization steps, 1,000 batches and 50,000 iterations per batch) for the microsatellite panel.

Population selfing rate, *s*, was derived from *g*_2_, estimated in *inbreedR* (Stoffel et al., 2016). *g*_2_ measures the extent to which heterozygosities are correlated across loci (David, Pujol, Viard, Castella, & Goudet, 2007). Under no inbreeding, the heterozygosities at different loci are expected to be statistically independent. The genetic data were permuted 1,000 times to test the hypothesis that the empirical *g*_2_ value is higher than the *g*_2_ for random associations between individuals and genotypes (i.e., *g*_2_ that is equal to 0). Selfing rate was estimated from *g*_2_ following equation 9 in David et al. (2007).

### 2.5 Spatial genetic structure (samples collected in 2015)

Assessment of population structure amongst the 510 RAD‐seq genotyped individuals collected in 2015 (originating from 11 natural habitats, 12 marinas and two cultivated populations) was performed using three different methods. First, we used discriminant analysis of principal components (DAPC), which is implemented in *adegenet* (Jombart & Ahmed, 2011). This method is able to refine the differentiation between populations while minimizing the within‐population differences. Second, we used a Bayesian clustering algorithm implemented in fastSTRUCTURE 1.0 (Raj, Stephens, & Pritchard, 2014). fastSTRUCTURE was run using default parameters, a simple prior and *K* (number of genetic clusters) values from 2 to 24. The analyses were then run with the logistic prior for the range of *K* plus one that was specified by the *chooseK.py* script. As *U. pinnatifida* is a partial selfer (see Results and Table 1), other clustering algorithms, such as the one implemented in INSTRUCT, which estimates individuals’ inbreeding coefficients while simultaneously grouping them into distinct genetic clusters (Gao, Williamson, & Bustamante, 2007), may perform better. However, INSTRUCT is based on a likelihood method and is, therefore, highly computationally intensive, which prevented us to analyse our RAD‐seq panel with this software. We instead used the computer program *snmf* developed by Frichot, Mathieu, Trouillon, Bouchard, and François (2014). Besides computational efficiency, *snmf* does not rely on Hardy–Weinberg equilibrium assumptions, in contrast to fastSTRUCTURE. It is thus particularly relevant to use in selfing species as shown by Frichot et al. (2014). Using simulated and empirical data sets, with the partial selfing species *Arabidopsis thaliana* as a case study, these authors showed that the *snmf* algorithm performs particularly well with high levels of inbreeding. The *snmf* algorithm is implemented as a function in the *LEA* package which has been optimized to process large population genomics data sets (Frichot & François, 2015). The *snmf* function was run with default parameters, with the number of iterations set to 1,000, and its outcomes were compared with those of fastSTRUCTURE. Admixture proportions for the optimal values of *K* were visualized in *pophelper* (Francis, 2017).

For the microsatellite panel, population structure was investigated amongst 789 individuals collected in 2015 utilizing DAPC and STRUCTURE version 2.3.4 (Falush, Stephens, & Pritchard, 2003). To estimate the most likely number of genetic clusters in the data set, *K*, 10 independent runs of *K = *1–25 were performed with 500,000 Markov chain Monte Carlo (MCMC) iterations following a 100,000 burn‐in period. No prior information specifying the definition of populations was entered into the model, which was run assuming correlated allele frequencies and admixture. Other parameters were left at default levels. The optimal value of *K* was chosen following the approach of Evanno, Regnaut, and Goudet (2005). We also compared the outcomes of STRUCTURE with those of INSTRUCT. We did not use the *snmf* algorithm because it cannot handle microsatellite loci with multiple allelic states. Similarly to STRUCTURE, INSTRUCT was run for *K* 1 to 25. We used default parameters, except that the number of independent chains for the MCMC algorithm was set to three.

To investigate the effect of habitat type, we performed a hierarchical analysis of molecular variance (AMOVA) in Arlequin 3.5.2.2 (Excoffier & Lischer, 2010), grouping the sampling localities by habitat type. The significance of covariance components and fixation indices was tested with 10,000 permutations. AMOVA was performed on the RAD‐seq panel only, with the M11‐15 samples excluded from the calculations due to low sample size.

The isolation‐by‐distance (IBD) model was tested on 22 natural habitat and marina localities, excluding M11‐15. Pairwise *F*_ST_ values were estimated with Arlequin, with the significance tested via 10,000 permutations and an alpha error set at 5%. Corrections for multiple comparisons were performed using the *p.adjust* function in R to control for the false discovery rate (FDR) (Benjamini & Hochberg, 1995). The genetic distance was Rousset's *F*_ST_/(1 −* F*_ST_) estimate for each pair of samples (Rousset, 1997). Geographic distance, in km, was estimated using the European coastline vector map (1:5000) (European Environment Agency) with ArcGis 10.4.1 (©Esfri). The significance of the IBD relationship was tested with a Mantel test in *vegan* (Oksanen et al., 2015) with 999 permutations. In addition, IBD was tested separately amongst the 11 natural habitat samples and amongst the 11 marina samples.

### 2.6 Temporal genetic structure (samples collected in 2005, 2009, 2015)

To investigate the importance of time versus habitat type in determining the overall genetic structure, we performed AMOVA on the five localities collected in 2005, 2009 and in 2015, and on M6 (sampled in 2005 and 2015). The samples were grouped by year within each habitat type. These analyses were performed in Arlequin using same parameters as detailed above. In addition, *F*_ST_ was estimated amongst the three temporal samples for each of the five localities. Significance was tested via 10,000 permutations. We then ran fastSTRUCTURE and *snmf* (RAD‐seq panel), as well as STRUCTURE and INSTRUCT (microsatellite panel), analyses with the 12 samples from the Bay of St. Malo (no. 12), using the same parameters as described above but testing for *K* between 1 and 12. In this bay, sampling was performed in 2005, 2009 and 2015 at all four study localities, which allows for fine‐scale investigation of spatial versus temporal drivers of *U*. *pinnatifida* genetic structure.

### 2.7 Genome scans (outlier detection)

With the aim of detecting outliers specific to marinas or natural habitats, we used a sample set (sample set 1) of 460 individuals originating from the 22 natural habitat and marina samples (excluding M11‐15) collected in 2015. Then, to also examine farms, we analysed a second sample set (sample set 2) of 186 individuals collected in 2015 from eight localities in the Bays of Morlaix and St. Malo with long‐standing farming activities (bays no. 8 and no. 12, respectively, in Figure 1). The two bays are replicates in terms of sampling strategy, with two natural samples, one marina and one farm sampled in each bay. Outlier detection was performed on 9,855 loci and on 7,550 loci for sample sets 1 and 2, respectively.

Because all outlier detection methods rely on specific assumptions, five methods were used with the RAD‐seq panel on both sample sets. First, we employed a Bayesian method that uses a logistic regression model to partition *F*_ST_ coefficients into a population‐specific component (beta) and a locus‐specific component (alpha), implemented in BayeScan 2.1 (Foll & Gaggiotti, 2008). Two other outlier tests were then carried out in Arlequin: the first is the default island model of Beaumont and Nichols (1996), whereas the second test utilizes a hierarchical island model that reduces the number of false‐positive outliers by accounting for population structure (Excoffier, Hofer, & Foll, 2009). For sample set 1, the individuals were grouped according to the genetic clusters identified by fastSTRUCTURE (see Results), and for sample set 2, the individuals were grouped by habitat type. Both methods were implemented with the default parameters. The fourth method was *pcadapt* 3.0.4 (Luu, Bazin, & Blum, 2017) that takes into account population structure (based on the principal component analysis). For both sample sets, the “mahalanobis” method was used to compute the *p*‐values. At last, we used OutFLANK (Whitlock & Lotterhos, 2015), which identifies outliers by comparing differentiation at each locus against a trimmed null distribution of *F*_ST_ values for loci that are deemed neutral. For sample set 1, OutFLANK was run with default parameters except that LeftTrimFraction = 0.4. For sample set 2, the programme was run with LeftTrimFraction = 0.7, RightTrimFraction = 0.1. Settings were chosen so as to improve the fit of the inferred neutral distribution of *F*_ST_. When needed, to correct for multiple tests, we used the FDR correction implemented in the R package *qvalue* (Storey, 2002). Only loci detected with three of four methods, excluding OutFLANK (see Results), were considered putative outliers.

With the microsatellite panel, the same two sample sets were analysed, corresponding to 725 individuals (including M11‐15) for sample sets 1 and 250 for sample set 2. Three of the five approaches described above, relevant for microsatellites, were used: BayeScan 2.1 with default parameters and Arlequin with the default and hierarchical island models.

## 3 RESULTS

### 3.1 Assembling and genotyping RAD‐seq loci

The sequencing run yielded 1,743,297,805 reads, of which 9.91% were dropped due to ambiguous barcodes. Of the 1,570,485,667 remaining reads, there were on average 2,074,618 reads per sample (with a standard deviation of 1,199,267). The number of reads did not differ substantially between samples from different years (on average 1,915,051 reads, 2,232,244 reads and 2,080,433 reads for the 2005, 2009 and 2015 samples, respectively). The Stacks catalogue was built from 1,105,640,102 reads remaining after filtering steps, with an average depth across all samples of 22.89 reads per locus. A total of 35,309 loci were present in at least 75% of the 757 individuals, of which 17,137 were polymorphic. Keeping only one randomly selected SNP per locus resulted in 14,622 SNPs. Following MAF selection, a total of 10,615 SNPs were used in subsequent analyses.

### 3.2 Comparison of genetic diversity and inbreeding amongst habitats

**Figure 2:**
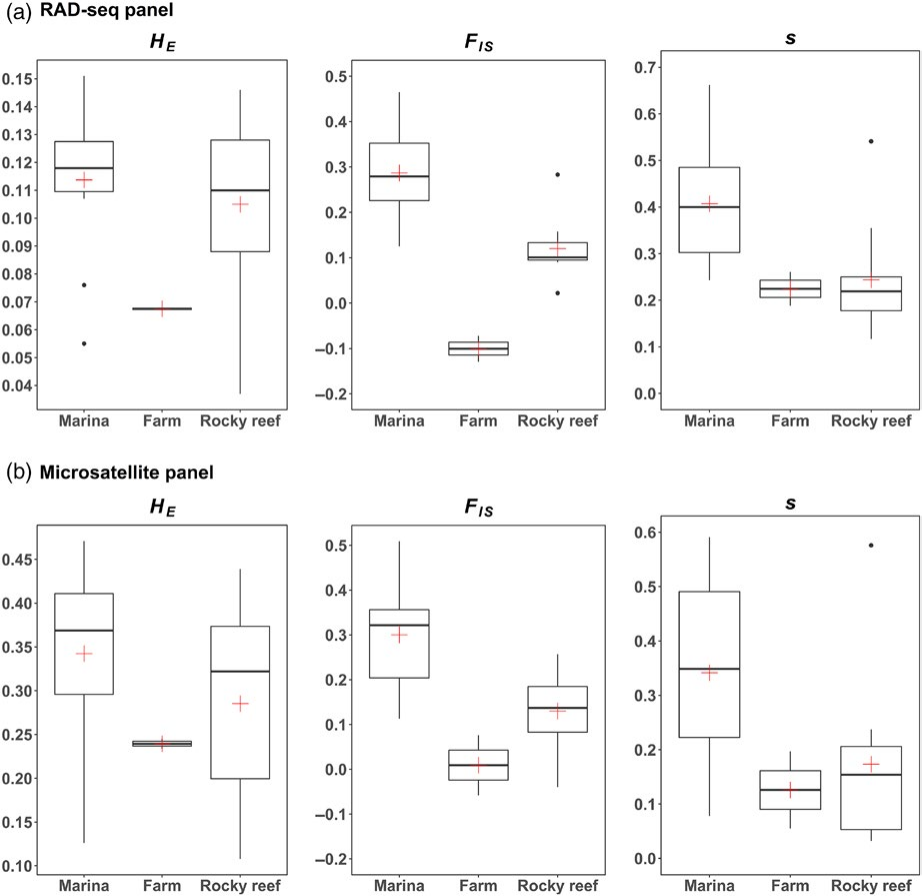
Comparison of genetic characteristics amongst habitat categories (marinas, farms, natural sites) in the 2015 data set for (a) RAD‐seq and (b) microsatellite markers. Boxplots indicating the average expected heterozygosity (*H*_*E*_), fixation index (*F*_*IS*_) and selfing rate estimate(s), with standard errors, for marinas (*N* = 11 and 12 for a and b, respectively), cultivated populations (*N* = 2), and natural rocky habitat sites (*N* = 11). The box shows the interquartile range (25–75th percentiles, with horizontal black line as median and red cross as mean). The upper and lower whiskers extend from the hinge to the largest and smallest value no further than 1.5 times the interquartile range. Data beyond this range (outliers) are plotted individually

For the RAD‐seq panel, over all the study samples (excluding M11‐15 with only two individuals), *H*_*e*_ ranged from 0.037 (in Rb8‐15) to 0.151 (in M12‐15) (Table 1). Of the three habitat types, the cultivated populations (farms) were the least genetically diverse. Levels of genetic diversity were two to three times higher in marinas and the rocky reef samples, as shown in Figure 2a for samples collected in 2015. Substantial variation was observed across samples for *F*_*IS*_, which ranged from −0.129 in F8‐15 to 0.465 in M2‐15, and for selfing rates, which varied from 0.052 to 0.662. Selfing was more prevalent in marinas than in natural reefs (Figure 2a). The microsatellite panel showed similar patterns (Supporting Information Table S1, Figure 2b).

### 3.3 Spatial genetic structure at the bay and regional scales

Microsatellite and RAD‐seq panels provided similar results. For example, the pairwise *F*_ST_ matrices for the RAD‐seq panel (Supporting Information Table S2A) and for microsatellites (Supporting Information Table S2B) were highly correlated (Mantel statistic *r* 0.790, *p*‐value 0.001). Therefore, below, we detail results obtained with the RAD‐seq panel only, except when microsatellites showed different results.

Overall, the 2015 populations were highly genetically structured at the regional scale and less so at the bay scale. For instance, the *F*_ST_ estimate computed over the four 2015 Bay of St. Malo localities was 0.166 (*p*‐value < 0.001), whereas the global *F*_ST_ for the 2015 samples was 0.313 (*p*‐value < 0.001). Pairwise *F*_ST_ comparisons amongst the 2015 samples revealed only three nonsignificant estimates (Supporting Information Table S2A), all of which were between natural and marina localities within the same bay (bays no. 3, 4 and 12; Figure 1). The rocky reef samples were slightly more genetically differentiated from one another (*F*_ST_ = 0.331, *p*‐value < 0.001) than were the marina samples (*F*_ST_ = 0.257, *p*‐value < 0.001). The two farms displayed slight, but significant (RAD‐seq panel only) pairwise genetic differentiation with each other and were highly genetically differentiated from all the other samples except for Ra8‐15 (Supporting Information Table S2A). Removing the two cultivated samples decreased the global 2015 *F*_ST_ to 0.291 (*p*‐value < 0.001). An AMOVA carried out on samples collected in 2015 with localities grouped according to habitat showed a low but significant difference amongst the three habitat types (*F*_CT_ *=* 0.046, *p*‐value < 0.001; Supporting Information Table S3), which disappeared when farms were removed from the analysis (i.e., two groups only, marinas and natural samples; *F*_CT_ = −0.011, *p*‐value 1.000; Supporting Information Table S3).

No IBD pattern was detected when analysing either the samples collected in natural reefs and marinas (*p*‐value 0.476), natural reefs only (*p*‐value 0.506) or marinas only (*p*‐value 0.829).

**Figure 3:**
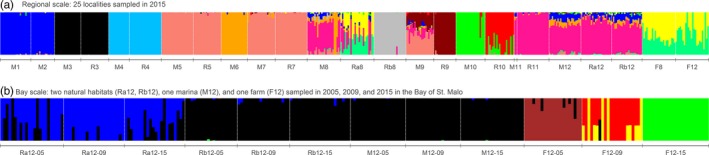
Bayesian clustering analyses (fastSTRUCTURE software) using the RAD‐seq panel (a) over the whole data set collected in 2015 and (b) in the Bay of St. Malo only (bay no. 12 in Figure 1). Each individual is represented by a vertical line divided into *K* coloured segments, the length of which indicates the individual's membership fraction to each of *K* clusters. Individuals are grouped according to their sampling locality (ordered along a south to north gradient) for the regional‐scale analysis, and according to locality and year of sampling for the analysis at the bay scale. Locality codes correspond to those given in Table 1

Without a priori knowledge on sampling localities, fastSTRUCTURE grouped the 510 individuals sampled in 2015 into 12 distinct genetic clusters (Figure 3a). In most cases (eight out of nine), samples originating from the marina and the rocky reef habitats sampled within the same bay were assigned to a single or a predominant cluster (bays no. 3, 4, 5, 7, 11, 12), or shared membership with one or two clusters found only in that bay (bays no. 9, 10). In bays no. 8 and 9, admixture was much more pronounced in marinas than in rocky reefs. The farm samples displayed a unique and specific genetic background, which was shared by some of the individuals sampled from only one rocky reef sample (Ra8‐15), which was very close (80 m) to the farm of the same bay (no. 8). The *LEA* cross‐entropy criterion suggested that the best *K* lies between 10 and 14. In an interesting manner, results obtained with the *snmf* function, shown in Supporting Information Figure S1A for *K* 12, are highly congruent with the results produced by fastSTRUCTURE. As the *snmf* algorithm does not rely on the Hardy–Weinberg equilibrium assumption, this result suggests that at the study regional scale, the variable, and sometimes high, selfing rates found in the study populations have little influence as compared to the spatial variation on the clustering performance by fastSTRUCTURE. The genetic proximity between marinas and rocky reefs from the same bay, as well as between certain geographically distant populations from similar habitat, was also supported by the DAPC (Supporting Information Figure S2A).

The microsatellite panel was less powerful in revealing genetic structure or assigning individuals to a specific cluster (Supporting Information Figure S2B). However, STRUCTURE analysis carried out on the microsatellite panel (Supporting Information Figure S3A) provided results broadly similar to those obtained with fastSTRUCTURE on the RAD‐seq panel (Figure 3a). In addition, as for the comparison between *snmf* and fastSTRUCTURE for the RAD‐seq panel, INSTRUCT (Supporting Information Figure S3B) showed results similar to those obtained with STRUCTURE (Supporting Information Figure S3A) for the microsatellite panel: in particular, the farms exhibited a distinctive pattern as compared to other populations, and some pairs of nearby marinas and natural reef localities displayed highly similar patterns. It is worth noting that overall the populations were less clearly distinguished from one another with INSTRUCT, using the microsatellite panel (Supporting Information Figure S3B), as compared to the results obtained using *snmf* with the RAD‐seq panel (Supporting Information Figure S1A).

### 3.4 Contrasting spatial and temporal patterns

**Figure 4:**
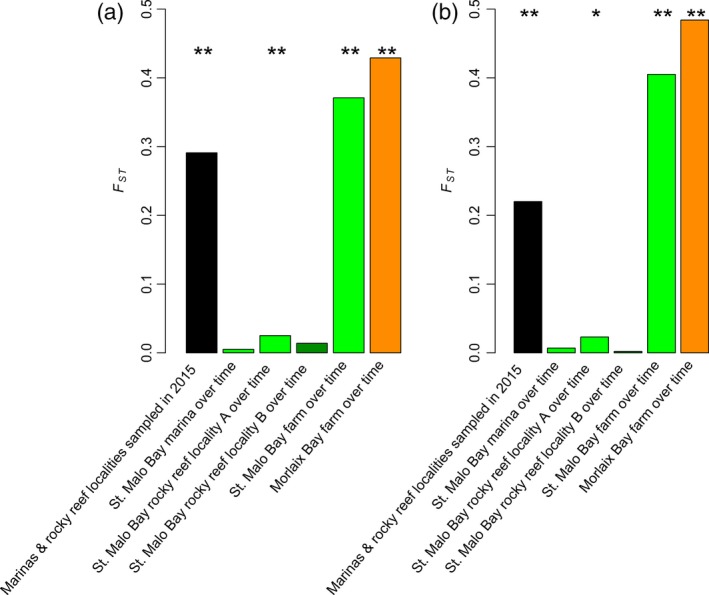
Spatial versus temporal genetic structure computed on (a) the RAD‐seq panel and (b) the microsatellite panel. Each bar represents the within‐group *F*_ST_, with the type of individuals comprising each group indicated on the *x*‐axis. *p‐value < 0.01, **p‐value < 0.001

Very little change was observed over time in the genetic composition of populations established in natural habitats and in marinas, especially compared with spatial variation (Figure 4). When localities were grouped per year, AMOVA showed that the genetic structure amongst years was non‐significant for natural reefs (*F*_CT_ = −0.013, *p*‐value 1.000) and marinas (*F*_CT_ = −0.062, *p*‐value 1.000) (Supporting Information Table S3). The result was very different in the cultivated populations, with large changes in the genetic composition of the crop amongst years (Figure 4, Supporting Information Table S3). Thus, spatial rather than temporal dynamics govern *U*. *pinnatifida* population genetic structure in natural habitats and marinas, but not in farms.

The fastSTRUCTURE and STRUCTURE analyses performed using samples from bay no. 12 (St. Malo) clearly confirmed this conclusion (Figure 3b for the RAD‐seq panel and Supporting Information Figure S3C for the microsatellite panel). Individuals sampled in farms clustered according to their year of sampling and were assigned to clusters different to those to which the marina or natural reef samples were assigned. On the contrary, the natural habitat and marina samples collected from different years were always assigned to the same cluster within a locality with the RAD‐seq panel (Figure 3b). A pattern of temporal stability was also observed with the microsatellite panel, although the localities were not distinguished from one another (Supporting Information Figure S3C). With *snmf* (RAD‐seq panel; Supporting Information Figure S1B) and INSTRUCT (microsatellite panel; Supporting Information Figure S3D), results similar to those obtained with fastSTRUCTURE (Figure 3b) and STRUCTURE (Supporting Information Figure S3C), respectively, were observed. This, again, suggests that selfing does not prevent reliable clustering with fastSTRUCTURE or STRUCTURE in the study area and species.

### 3.5 Outlier detection

With sample set 1, comprising 460 individuals collected in 2015 from 22 natural rocky reef and marina samples spanning two biogeographical provinces, 240 RAD‐seq loci (2.44% of the investigated loci) were identified as outliers across all methods (Supporting Information Figure S4A). OutFLANK did not detect any positive selection outliers at a *q*‐value of 0.05. A single locus was detected as an outlier by all four remaining approaches (Supporting Information Figure S4A). In an interesting manner, OutFLANK also identified this locus as an outlier at a *q*‐value of 0.056. This locus was monomorphic in all localities except the two localities sampled in bay no. 4, where it was monomorphic for an alternative allele. Examining all the outliers identified by at least three of the four methods (excluding OutFLANK) revealed that these loci were specific to a bay rather than to a particular habitat. For OutFLANK, a conservative approach, we examined the 64 loci detected at a *q*‐value of 0.056: 40 loci behaved as outliers within a single locality—Men Guen (Rb8‐15 in Figure 1) and the remaining 24 in bay no. 4. Thus, these loci were acting as outliers within a specific bay and not within a specific habitat or region, which was also found with the other approaches. These “locality” singularities are pictured with a fastSTRUCTURE analysis in Supporting Information Figure S5A. Note that removing the 240 outliers detected across all methods did not differ from the results obtained with the full RAD‐seq panel (see an example for fastSTRUCTURE analysis in Supporting Information Figure S5B). None of the microsatellite panel loci were detected as outliers by any of the three methods for sample set 1.

With sample set 2, which was used to compare the three habitats (i.e., two bays, each with three habitat categories), 294 (3.89% of the investigated loci) outliers were found across all methods. None of the BayeScan‐identified outliers were detected by any other method (Supporting Information Figure S4B). A total of 59 loci (0.78% of the 7,550 loci examined) were detected by the three other methods, excluding OutFLANK which did not detect any loci under positive selection at *q*‐value of 0.05. Fifty‐eight of these loci, also detected as outliers by OutFLANK but at a *q*‐value of 0.141, pointed to the Men Guen sample (Rb8‐15). In an interesting manner, the remaining locus was fixed for an alternative allele only in the two study farms. Nine other loci, identified as outliers with two methods, also discriminated the two farms. For the microsatellite panel, one locus was identified as an outlier by two of the three methods but without any particular pattern.

## 4 DISCUSSION

Despite the acute need for improved understanding of the evolutionary dynamics of marine invasions, notably for setting up more effective management and for curtailing the invasion rates, few studies have examined population genomics of NIS, and most have focused on issues related to hybridization between lineages (e.g., *Carcinus maenas*, Jeffery et al., 2017) or species (e.g., *Mytilus* spp. Saarman & Pogson, 2015), or for comparing native and non‐native populations (e.g., *Crassostrea* spp., Gagnaire et al., 2018). Our study focused on the post‐introduction colonization dynamics of one marine NIS, the invasive kelp *U. pinnatifida,* with the specific aim to understand the relationships between populations established in the primary sites of introduction (farms and marinas) and the nearby natural reefs to which the species had spread, and determine whether some location adaptation had occurred. Our results revealed: (a) contrasting genetic properties linked to habitat; (b) absence of isolation by distance; (c) stability of the genetic composition of populations in natural habitat over ca. 20 generations; and (d) no specific signatures of adaptation to habitat type. These main findings are discussed below in the light of our initial hypotheses, which were only partially confirmed.

### 4.1 Contrasted genetic properties amongst habitat types, particularly the farms

The populations sampled from the three habitat types exhibited habitat‐specific genetic properties. Other marine species have shown differences amongst populations inhabiting different habitat types. For example, differences were found between natural reefs and hatcheries in the Manila clam *Ruditapes philippinarum* (Xing, Gao, & Li, 2014), between enclosed and open marinas in the invasive tunicate *Styela clava* (Dupont, Viard, Dowell, Wood, & Bishop, 2009), or between fixed and free‐floating populations of the invasive red seaweed *Gracilaria vermiculophylla* (Krueger‐Hadfield et al., 2016). Here, farmed populations were highly distinct from the other types of populations because, for a given sampling year, they displayed the lowest genetic diversity, and showed no inbreeding signal (negative F_IS_, Figure 2a). In addition, the two farm samples were the only ones to show temporal instability (Figures 3b and 4a). Pooling the three farm samples from St. Malo increased the gene diversity (*H*_*e*_ = 0.130) to a level similar to that found in 2015 in many marina or natural reefs (Table 1).

These results indicate that the cultivated stock origin varied amongst years. These genetic patterns are consistent with known farming practices. Each year the farmers produce new germlines from just a few healthy and mature individuals sampled on culture lines and/or from the wild. This practice results in considerable sampling and genetic drift from one year to the next, explaining both the low diversity and the temporal changes in the genetic composition of the cultivated populations. The farmers also actively outcross their stock by mixing female and male gametophytes obtained from different individuals, thus minimizing inbreeding. Although selection through domestication has been documented in the native range of *U. pinnatifida* (Valero et al., 2017), we have no evidence of an effect of the farming practices in the study non‐native range. We found only one outlier locus specific to the two farms sampled in 2015. However, the owners of these two farms informed us that they used the same germlines that year, meaning that our two farm samples were technical rather than biological replicates. Further studies (e.g., QTL‐based) are needed to examine to what extent farming practices promote specific adaptation of cultivated individuals. This QTL strategy was efficiently used in farmed Atlantic salmon to document the discovery of a major QTL affecting resistance to a viral disease (Houston et al., 2012). Nevertheless, our study unambiguously documented that the genetic properties of the farm samples can be fully explained by the farming practices and are in clear contrast to the genetic properties of the samples inhabiting the marinas and natural habitats.

Populations sampled in marinas and in natural habitats displayed roughly equivalent levels of genetic diversity (Figure 2). In some bays, marinas and natural reefs were however different from one another regarding the extent of admixture which was more pronounced in marinas (Figure 3a), a finding that sheds light on interpopulation connectivity (see below). Similar conclusions were reached with microsatellites in a previous study at a much smaller spatial scale (one bay), but on a much larger number of natural reefs (Grulois et al., 2011). The levels of inbreeding were also particularly high, both in marinas and in natural reefs (although higher in the former), and explained by extremely high selfing rates. This concurs well with the previous findings in the native and introduced range that *U*. *pinnatifida* is not only self‐compatible, but also an efficient selfer. This life‐history trait confers a major advantage to colonist and pioneer species, including NIS, enabling rapid local spread and population growth (Sakai et al., 2001). Uniparental reproduction, through selfing or asexual reproduction, has been shown to facilitate colonization of new habitats in terrestrial plants (Barrett, 2011; Pannell et al., 2015). On several occasions, it has also been pointed out as an important trait in invasive seaweeds. For instance, in its North American and European introduction ranges, unlike in its native range, the red alga *Gracilaria vermicullophyta* was shown to display a shift towards a higher ratio of diploid individuals associated with a transition from sexual to asexual reproduction (Krueger‐Hadfield et al., 2016). The ability to self‐fertilize is also one characteristic listed amongst other traits to explain the high invasion success of the brown seaweed *Sargassum muticum,* a circumglobal invader (Engelen et al., 2015). As suggested by Nyberg and Wallentinius (2005), uniparental reproduction could be an important correlate of invasive success in seaweeds, although more detailed comparative analyses between invasive and noninvasive species or between native and introduced populations are required to ascertain this hypothesis.

### 4.2 Human‐mediated dispersal is still responsible for large‐scale connectivity

At the regional (i.e., Brittany) scale, *U*. *pinnatifida* populations were highly structured genetically, but not in accordance with the habitat, geographic distance or biogeographical region. As shown by the fastSTRUCTURE plot (Figure 3a) and the DAPC (Supporting Information Figure S2A), there was high genetic similarity between samples occupying distant localities, such as bays no. 8 (Bay of Morlaix) and no. 12 (Bay of St. Malo) (Figure 1), but clear separation of samples collected from nearby bays, like the Etel sample (bay no. 3) and bays no. 1 (La Trinité‐Sur‐Mer) and no. 2 (Quiberon). This pattern cannot be explained by natural dispersal by spores and gametes which are short‐lived, and thus expected to drive dispersal over short distances only (1–100 m) (Forrest et al., 2000). Drifting thalli detached from substrate after storms or attached to unstable substrate, such as pebbles, might be responsible for longer dispersal distances (1–10 km) (Forrest et al., 2000; Sliwa et al., 2006), and, thus, could explain spread within a bay or between neighbouring bays (Grulois, 2010; Grulois et al., 2011). However, these natural dispersal means are unlikely to explain spread over more than 10–100 km, as suggested here by the close genetic proximity between distant localities, and the overall chaotic genetic pattern observed at a regional scale. This chaotic pattern is very likely driven by random human‐mediated dispersal rather than by more or less stepwise natural dispersal of *U*. *pinnatifida*. The absence of IBD also lends support to our hypothesis of human‐mediated transport exerting strong influence on the genetic structure of *U*. *pinnatifida* at a regional scale. Similar chaotic connectivity patterns have been found in other marine NIS, such as the tunicate *Styela clava* (Dupont et al., 2009; Goldstien, Schiel, & Gemmell, 2010), as well as in native species established in marinas like the ascidian *Ciona intestinalis* (Hudson, Viard, Roby, & Rius, 2016), associated with boating activities. Floating pontoons and leisure boats, docking in the marinas, etc., are providing new habitats to many and diverse NIS taxa (e.g., bryozoans (Marchini, Ferrario, & Minchin, 2015), caprellids (Ros et al., 2013)) and are pathways of NIS spread (Clarke Murray, Pakhomov, & Therriault, 2011; Mineur, Johnson, & Maggs, 2008). Young or mature *U*. *pinnatifida* sporophytes are regularly observed attached to boats docking in the studied marinas (authors, personal observation), and microscopic gametophytes are likely to be transported by various means (ropes, hull fouling, ballast water, etc.) via boating activities (Epstein & Smale, 2017b). Human‐mediated spread, notably through shipping (commercial, fishing or leisure boats), has been pointed out as a main vector of spread in Australasia (South et al., 2017, and references herein). Similar to that, based on our study, in Brittany, 40 years after its introduction, the overall connectivity pattern of *U. pinnatifida* at a regional scale appears to be still largely driven by the effects of human‐mediated dispersal, notably through leisure boat movements.

### 4.3 Spillover effects from marinas but not from farms

Some preliminary insights regarding relationships amongst habitats of *U*. *pinnatifida* came from a combined field and microsatellite study carried out at a local scale (i.e., within one bay, the Bay of St. Malo; Grulois et al., 2011). *U*. *pinnatifida* specimens were found at 84% of the 37 prospected sites. Based on their genetic results, marinas—a transit zone hosting leisure boats that have potentially visited many different localities—appeared to be the main source of propagule introduction into natural rocky reefs. Our findings considerably expand these preliminary conclusions: we detected similar connectivity in most studied bays, and across a regional scale (along ca. 1,000 km of coastline). The marina and rocky reef populations sampled in the same bay were frequently genetically very similar: they were assigned to the same genetic cluster(s) by fastSTRUCTURE (Figure 3a), displayed low pairwise *F*_ST_ values (sometimes nonsignificant; Supporting Information Table S2), and clustered in the DAPC (Supporting Information Figure S2A). In addition, the lack of an IBD signal across the natural reefs only suggested that human‐mediated dispersal predominates dispersal by natural means through spores and gametes (which would be expected to occur in a stepwise manner). This lack of IBD amongst rocky reefs colonized by *U*. *pinnatifida,* in its introduction range*,* is indeed in contrast with results obtained from a meta‐analysis carried out on 21 native macroalgal species, including 16 brown seaweeds, that found evidence of significant IBD whatever the habitat (intertidal or subtidal) or life cycle (Durrant et al., 2014). The absence of IBD in the two types of habitat also gives further credence to the close link between the marina and nearby rocky reefs, thus providing evidence for spillover effects from marinas into the wild habitats, as suggested by recent field surveys carried out in the same region (i.e., the English Channel; Epstein & Smale, 2017a).

Compared with the close relationship we documented between the marinas and the nearby natural reefs, the cultivated populations were genetically differentiated from all the natural reefs studied, with only one exception, that is, the reef located very near to one of the farms (Ra8) in bay no. 8 (St. Malo). However, the other natural population studied in this bay (Rb8) did not show such high similarity with the farm. Overall, there was no evidence that cultivated populations are contributing (i.e., due to escapes from the farms) to the immigrant pool reaching rocky reefs or marinas. It is assumed that at the very beginning of *U*. *pinnatifida* colonization of the European Atlantic coastline, the escapees from the cultivated sites were the main source of recruits into surrounding hard substrates (marinas or rocky reef habitats; Floc'h et al., 1996), and this was supported by a worldwide genetic study (Voisin et al., 2005). The high genetic diversity observed in the studied marinas, however, also suggests that this deliberate primary introduction in Brittany may not be the only primary source of the introduction in the region. In addition, our results indicate that, after the first few generations, farms stopped providing large numbers of effective migrants into these populations. This may be due to either tighter controls exerted by the farmers that were preventing more individuals from “escaping,” the decreasing number of farms, as some were shut down after the first trials, or alternative propagule sources. Whatever the cause, our results suggest diminished propagule pressure from farms since this species was first introduced to and became established in the wild (i.e., since the 1980s). Our results also strongly suggest that marinas are currently the most important source of immigrants in natural rocky reefs. Density effects likely play an important role together with other factors, such as the structure of the native macroalgal canopy or wave exposure (Epstein & Smale, 2017a; James & Shears, 2016).

### 4.4 Self‐sustaining populations in marinas and natural habitats and implications for local adaptation

We showed that populations in marinas and rocky reefs behave as self‐sustaining populations in the Bay of St. Malo. The marinas and natural reefs were indeed highly genetically stable over time, as indicated by AMOVA, global *F*_ST_ and fastSTRUCTURE analyses carried out on samples collected in 2005, 2009 and 2015 (i.e., over roughly 20 generations). This self‐sustaining scenario has been confirmed in a microsatellite‐based genetic survey of the colonization dynamics of a newly built marina (Salomon, M., Lévêque, L., Ballenghien, M. & Viard, F., unpublished data). With limited dispersal ability, local recruitment is essential for long‐term maintenance of the population as commonly reported in native kelps (Valero et al., 2011), and here in a non‐native kelp. The joint effect of high selfing rates, short reproductive period and intraspecific competition may explain the low influence of putative immigrants in the study kelp.

Despite populations apparently closed to immigration, no signatures of local adaptation to a particular habitat type were detected. Several causes may be behind the fact that only few outliers were detected and none were linked to habitats: (a) the methodology itself, as genome scans have limited efficiency, except if few genes with strong effects are involved; (b) insufficient lapse of time since interhabitat gene flow ceased; (c) the persistence of *U*. *pinnatifida* in diverse environments due to acclimatization rather than adaptation; and (d) our coarse‐grained classification of the populations. These hypotheses are not mutually exclusive. The traits that may confer an advantage in the different study environments are likely determined by many genes (e.g., in relation with complex traits like life cycle duration, reproduction, etc.). In addition, NIS are known to display substantial levels of phenotypic plasticity (Davidson, Jennions, & Nicotra, 2011), a likely mechanism in *U. pinnatifida* (Murphy et al., 2017). Rapid phenotypic evolution in non‐native populations of the red alga *Gracilaria vermiculophylla* has been recently reported by Sotka et al. (2018), and should be better examined in *U. pinnatifida*. At last, it is interesting to note that the few outliers identified singled out specific localities, namely the two populations sampled in bay no. 4 (Loctudy) and the Men Guen population (Rb8) sampled in bay no. 8 (Morlaix). For bay no. 4, this pattern could be due to a singular history of introduction, because a new large farm was recently established in this area. The singularity of bay no. 4 in the neutral RAD‐seq panel supports this hypothesis. Regarding sample Rb8, the hypothesis of a specific environmental feature is interesting to consider: this locality, where the species was reported for the first time 10 years ago, is exposed to strong waves, which is a rare habitat for *U. pinnatifida*. Further dedicated studies are needed, to characterize the fine‐grained environmental conditions (e.g., wave exposure, nutrients availability, irradiance) using local autonomous data loggers in the areas colonized by *U. pinnatifida* and then examine genomic‐environmental associations, as recently done in the invasive crab *Carcinus maenas* (Jeffery et al., 2018). In addition, a genome sequencing project is currently in progress (Yoon et al., pers. comm.). Once completed, comparison with the RAD‐seq panel based genome‐wide investigation can be used, for instance, to map the outliers and determine whether they are clustered and associated with specific genes or functions.

### 4.5 Conclusion

The results of the present study were based on over ca. 10,000 SNPs derived from ddRAD‐sequencing. As expected (Gagnaire et al., 2015), the RAD‐seq panel showed more power than the microsatellite panel for identifying fine‐grained genetic structure patterns, as well as for detecting outliers. However, overall, both marker panels revealed similar patterns. This congruency gave credence to the bioinformatic assembling and filtering steps performed on ddRAD‐sequencing data. It also upholds the value of microsatellite markers for future studies, particularly in the absence of a reference genome. This study provided evidence for our hypothesis of the persistent effect of human‐mediated transport, such as boating activities, on connectivity of *U*. *pinnatifida* at a regional scale. Whereas farms were the primary source of the introduction of *U*. *pinnatifida* in the study area, our results clearly support the hypothesis that present‐day spillover effects from farms are negligible, compared with from marinas. We also showed that after colonization, populations eventually become self‐sustaining. Although we did not capture signatures of local adaptation after only 20–40 generations following the foundation of wild populations, we anticipate that adaptation can nevertheless occur if the populations remain self‐sustaining and protected from gene flow counterbalancing local adaptation to their local natural environment. Under the current situation in which farming activities are maintained at low level, our results demonstrate the need for management targeting marinas to reduce the risk of spread of *U*. *pinnatifida* into the natural environment. This is especially important for newly built marinas, or marinas not yet colonized by *U*. *pinnatifida*, particularly those that occur in areas where the surrounding rocky reefs are still free of this kelp. Tight controls on the (bio)fouling of the floating pontoons and of hulls of boats docking there should be established. Such management measures would be helpful to prevent spread beyond the study species as spillover from marinas might occur for other NIS of the fouling community.

## CONFLICT OF INTEREST

None declared

## DATA AVAILABILITY

ddRAD‐seq and microsatellite data set are available as a VCF file and a multilocus genotype file uploaded on Dryad Digital Repository at https://doi.org/10.5061/dryad.cd4q0ds.

## References

[eva12647-bib-0001] Airoldi, L. , Turon, X. , Perkol‐Finkel, S. , & Rius, M. (2015). Corridors for aliens but not for natives: Effects of marine urban sprawl at a regional scale. Diversity and Distributions, 21(7), 755–768. 10.1111/ddi.12301

[eva12647-bib-0002] Araújo, R. , Assis, J. , Aguillar, R. , Airoldi, L. , Bárbara, I. , Bartsch, I. , … Derrien‐Courtel, S. (2016). Status, trends and drivers of kelp forests in Europe: An expert assessment. Biodiversity and Conservation, 25(7), 1319–1348. 10.1007/s10531-016-1141-7

[eva12647-bib-0003] Barrett, S. C. H. (2011). Why reproductive systems matter for the invasion biology of plants In Richardson D. (Ed.), Fifty years of invasion ecology: The legacy of Charles Elton (pp. 195–210). Oxford: Wiley‐Blackwell.

[eva12647-bib-0004] Beaumont, M. A. , & Nichols, R. A. (1996). Evaluating loci for use in the genetic analysis of population structure. Proceedings of the Royal Society of London B: Biological Sciences, 263(1377), 1619–1626. 10.1098/rspb.1996.0237

[eva12647-bib-0005] Benjamini, Y. , & Hochberg, Y. (1995). Controlling the false discovery rate: A practical and powerful approach to multiple testing. Journal of the Royal Statistical Society: Series B (Methodological), 57, 289–300.

[eva12647-bib-0006] Bierne, N. , Welch, J. , Loire, E. , Bonhomme, F. , & David, P. (2011). The coupling hypothesis: Why genome scans may fail to map local adaptation genes. Molecular Ecology, 20(10), 2044–2072. 10.1111/j.1365-294X.2011.05080.x 21476991

[eva12647-bib-0007] Bishop, M. J. , Mayer‐Pinto, M. , Airoldi, L. , Firth, L. B. , Morris, R. L. , Loke, L. H. L. , … Dafforn, K. A. (2017). Effects of ocean sprawl on ecological connectivity: Impacts and solutions. Journal of Experimental Marine Biology and Ecology, 492, 7–30. 10.1016/j.jembe.2017.01.021

[eva12647-bib-0008] Bishop, J. , Wood, C. A. , Lévêque, L. , Yunnie, A. L. E. , & Viard, F. (2015). Repeated rapid assessment surveys reveal contrasting trends in occupancy of marinas by non‐indigenous species on opposite sides of the western English Channel. Marine Pollution Bulletin, 95, 699–706. 10.1016/j.marpolbul.2014.11.043 25534627

[eva12647-bib-0009] Bock, D. G. , Caseys, C. , Cousens, R. D. , Hahn, M. A. , Heredia, S. M. , Hübner, S. , … Rieseberg, L. H. (2015). What we still don't know about invasion genetics. Molecular Ecology, 24(9), 2277–2297. 10.1111/mec.13032 25474505

[eva12647-bib-0010] Brelsford, A. , Dufresnes, C. , & Perrin, N. (2016). High‐density sex‐specific linkage maps of a European tree frog (*Hyla arborea*) identify the sex chromosome without information on offspring sex. Heredity, 116(2), 177–181. 10.1038/hdy.2015.83 26374238 PMC4806884

[eva12647-bib-0011] Bulleri, F. , & Chapman, M. G. (2010). The introduction of coastal infrastructure as a driver of change in marine environments. Journal of Applied Ecology, 47(1), 26–35. 10.1111/j.1365-2664.2009.01751.x

[eva12647-bib-0012] Capinha, C. , Essl, F. , Seebens, H. , Moser, D. , & Pereira, H. M. (2015). The dispersal of alien species redefines biogeography in the Anthropocene. Science, 348(6240), 1248–1251. 10.1126/science.aaa8913 26068851

[eva12647-bib-0013] Castric‐Fey, A. , Beaupoil, C. , Bouchain, J. , Pradier, E. , & L'Hardy‐Halos, M. T. (1999). The introduced alga *Undaria pinnatifida* (Laminariales, Alariaceae) in the rocky shore ecosystem of the St Malo area: Morphology and growth of the sporophyte. Botanica Marina, 42(1), 71–82.

[eva12647-bib-0014] Castric‐Fey, A. , Girard, A. , & L'Hardy‐Halos, M. T. (1993). The distribution of *Undaria pinnatifida* (Phaeophyceae, Laminariales) on the coast of St. Malo (Brittany, France). Botanica Marina, 36(4), 351–358.

[eva12647-bib-0015] Catchen, J. , Hohenlohe, P. A. , Bassham, S. , Amores, A. , & Cresko, W. A. (2013). Stacks: An analysis tool set for population genomics. Molecular Ecology, 22(11), 3124–3140. 10.1111/mec.12354 23701397 PMC3936987

[eva12647-bib-0016] Clarke Murray, C. , Pakhomov, E. A. , & Therriault, T. W. (2011). Recreational boating: A large unregulated vector transporting marine invasive species. Diversity and Distributions, 17(6), 1161–1172. 10.1111/j.1472-4642.2011.00798.x

[eva12647-bib-0017] Costello, M. J. , Bouchet, P. , Boxshall, G. , Fauchald, K. , Gordon, D. , Hoeksema, B. W. , … Appeltans, W. (2013). Global coordination and standardisation in marine biodiversity through the World Register of Marine Species (WoRMS) and Related Databases. PLoS ONE, 8(1), e51629 10.1371/journal.pone.0051629 23505408 PMC3541386

[eva12647-bib-0019] Daguin, C. , Voisin, M. , Engel, C. , & Viard, F. (2005). Microsatellites isolation and polymorphism in introduced populations of the cultivated seaweed *Undaria pinnatifida* (Phaeophyceae, Laminariales). Conservation Genetics, 6(4), 647–650.

[eva12647-bib-0020] Darling, J. A. , Galil, B. S. , Carvalho, G. R. , Rius, M. , Viard, F. , & Piraino, S. (2017). Recommendations for developing and applying genetic tools to assess and manage biological invasions in marine ecosystems. Marine Policy, 85, 54–64. 10.1016/j.marpol.2017.08.014 PMC5909192 29681680

[eva12647-bib-0021] David, P. , Pujol, B. , Viard, F. , Castella, V. , & Goudet, J. (2007). Reliable selfing rate estimates from imperfect population genetic data. Molecular Ecology, 16(12), 2474–2487. 10.1111/j.1365-294X.2007.03330.x 17561907

[eva12647-bib-0022] Davidson, A. M. , Jennions, M. , & Nicotra, A. B. (2011). Do invasive species show higher phenotypic plasticity than native species and if so, is it adaptive? A meta‐analysis Ecology Letters, 14(4), 419–431. 10.1111/j.1461-0248.2011.01596.x 21314880

[eva12647-bib-0023] De Leij, R. , Epstein, G. , Brown, M. P. , & Smale, D. A. (2017). The influence of native macroalgal canopies on the distribution and abundance of the non‐native kelp *Undaria pinnatifida* in natural reef habitats. Marine Biology, 164(7), 156 10.1007/s00227-017-3183-0

[eva12647-bib-0024] Dupont, L. , Viard, F. , Dowell, M. , Wood, C. , & Bishop, J. (2009). Fine‐and regional‐scale genetic structure of the exotic ascidian *Styela clava* (Tunicata) in southwest England, 50 years after its introduction. Molecular Ecology, 18(3), 442–453. 10.1111/j.1365-294X.2008.04045.x 19161467

[eva12647-bib-0025] Durrant, H. , Burridge, C. P. , Kelaher, B. P. , Barrett, N. S. , Edgar, G. J. , & Coleman, M. A. (2014). Implications of macroalgal isolation by distance for networks of marine protected areas. Conservation biology, 28(2), 438–445. 10.1111/cobi.12203 24373031

[eva12647-bib-0026] Engelen, A. H. , Serebryakova, A. , Ang, P. , Britton‐Simmons, K. , Mineur, F. , Pedersen, M. , … Santos, R. (2015). Global invasion by the brown seaweed *Sargassum muticum* . Oceanography and Marine Biology: An Annual Review, 53, 81–126.

[eva12647-bib-0027] Epstein, G. , & Smale, D. A. (2017a). Environmental and ecological factors influencing the spillover of the non‐native kelp, *Undaria pinnatifida*, from marinas into natural rocky reef communities. Biological Invasions, 20, 1049–1072. 10.1007/s10530-017-1610-2 PMC6560939 31258384

[eva12647-bib-0028] Epstein, G. , & Smale, D. A. (2017b). *Undaria pinnatifida*: A case study to highlight challenges in marine invasion ecology and management. Ecology and Evolution., 7(20), 8624–8642. 10.1002/ece3.3430 29075477 PMC5648660

[eva12647-bib-0029] Evanno, G. , Regnaut, S. , & Goudet, J. (2005). Detecting the number of clusters of individuals using the software STRUCTURE: A simulation study. Molecular Ecology, 14(8), 2611–2620. 10.1111/j.1365-294X.2005.02553.x 15969739

[eva12647-bib-0030] Excoffier, L. , Hofer, T. , & Foll, M. (2009). Detecting loci under selection in a hierarchically structured population. Heredity, 103(4), 285–298. 10.1038/hdy.2009.74 19623208

[eva12647-bib-0031] Excoffier, L. , & Lischer, H. E. (2010). Arlequin suite ver 3.5: A new series of programs to perform population genetics analyses under Linux and Windows. Molecular ecology resources, 10(3), 564–567. 10.1111/j.1755-0998.2010.02847.x 21565059

[eva12647-bib-0032] Falush, D. , Stephens, M. , & Pritchard, J. K. (2003). Inference of population structure using multilocus genotype data: Linked loci and correlated allele frequencies. Genetics, 164(4), 1567–1587. 12930761 10.1093/genetics/164.4.1567 PMC1462648

[eva12647-bib-0033] Fauvelot, C. , Bertozzi, F. , Costantini, F. , Airoldi, L. , & Abbiati, M. (2009). Lower genetic diversity in the limpet *Patella caerulea* on urban coastal structures compared to natural rocky habitats. Marine Biology, 156(11), 2313–2323. 10.1007/s00227-009-1259-1

[eva12647-bib-0034] Fisher, A. C. , Volpe, J. P. , & Fisher, J. T. (2014). Occupancy dynamics of escaped farmed Atlantic salmon in Canadian Pacific coastal salmon streams: Implications for sustained invasions. Biological invasions, 16(10), 2137–2146. 10.1007/s10530-014-0653-x

[eva12647-bib-0035] Fletcher, R. , & Farrell, P. (1999). Introduced brown algae in the North East Atlantic, with particular respect to *Undaria pinnatifida* (Harvey) Suringar. Helgoländer Meeresuntersuchungen, 52(3), 259.

[eva12647-bib-0036] Floc'h, J.‐Y. , Pajot, R. , & Mouret, V. (1996). *Undaria pinnatifida* (Laminariales, Phaeophyta) 12 years after its introduction into the Atlantic Ocean. Hydrobiologia, 326(1), 217–222. 10.1007/BF00047810

[eva12647-bib-0037] Foll, M. , & Gaggiotti, O. (2008). A genome‐scan method to identify selected loci appropriate for both dominant and codominant markers: A Bayesian perspective. Genetics, 180(2), 977–993. 10.1534/genetics.108.092221 18780740 PMC2567396

[eva12647-bib-0038] Forrest, B. M. , Brown, S. N. , Taylor, M. D. , Hurd, C. L. , & Hay, C. H. (2000). The role natural dispersal mechanisms in the spread of *Undaria pinnatifida* (Laminariales, Phaeophyceae). Phycologia, 39(6), 547–553. 10.2216/i0031-8884-39-6-547.1

[eva12647-bib-0039] Francis, R. M. (2017). Pophelper: An R package and web app to analyse and visualize population structure. Molecular Ecology Resources, 17(1), 27–32. 10.1111/1755-0998.12509 26850166

[eva12647-bib-0040] Frichot, E. , & François, O. (2015). LEA: An R package for landscape and ecological association studies. Methods in Ecology and Evolution, 6(8), 925–929. 10.1111/2041-210X.12382

[eva12647-bib-0041] Frichot, E. , Mathieu, F. , Trouillon, T. , Bouchard, G. , & François, O. (2014). Fast and efficient estimation of individual ancestry coefficients. Genetics, 196(4), 973–983. 10.1534/genetics.113.160572 24496008 PMC3982712

[eva12647-bib-0042] Gagnaire, P. A. , Broquet, T. , Aurelle, D. , Viard, F. , Souissi, A. , Bonhomme, F. , … Bierne, N. (2015). Using neutral, selected, and hitchhiker loci to assess connectivity of marine populations in the genomic era. Evolutionary Applications, 8(8), 769–786. 10.1111/eva.12288 26366195 PMC4561567

[eva12647-bib-0043] Gagnaire, P.‐A. , Lamy, J.‐B. , Cornette, F. , Heurtebise, S. , Degremont, L. , Flahauw, E. , … Lapegue, S. (2018). Analysis of genome‐wide differentiation between native and introduced populations of the cupped oysters *Crassostrea gigas* and *Crassostrea angulata* . 10.1101/292144 PMC6161763 30184067

[eva12647-bib-0044] Gao, H. , Williamson, S. , & Bustamante, C. D. (2007). A Marko Chain Monte Carlo approach for joint inference of population structure and inbreeding rates from multi‐locus genotype data. Genetics, 76, 1635–1651. 10.1534/genetics.107.072371 PMC1931536 17483417

[eva12647-bib-0045] Glasby, T. , Connell, S. , Holloway, M. , & Hewitt, C. (2007). Nonindigenous biota on artificial structures: Could habitat creation facilitate biological invasions? Marine Biology, 151(3), 887–895. 10.1007/s00227-006-0552-5

[eva12647-bib-0046] Glover, K. A. , Solberg, M. F. , McGinnity, P. , Hindar, K. , Verspoor, E. , Coulson, M. W. , … Svåsand, T. (2017). Half a century of genetic interaction between farmed and wild Atlantic salmon: Status of knowledge and unanswered questions. Fish and Fisheries, 18(5), 890–927. 10.1111/faf.12214

[eva12647-bib-0047] Goldstien, S. , Schiel, D. , & Gemmell, N. (2010). Regional connectivity and coastal expansion: Differentiating pre‐border and post‐border vectors for the invasive tunicate Styela clava. Molecular Ecology, 19(5), 874–885. 10.1111/j.1365-294X.2010.04527.x 20149095

[eva12647-bib-0048] Gosselin, T. , & Bernatchez, L. (2016). stackr: GBS/RAD Data Exploration, Manipulation and Visualization using R. R package version 0.2.

[eva12647-bib-0049] Grulois, D. (2010). Etude de la dispersion et du recrutement à différentes échelles spatiales chez Undaria pinnatifida, une macro‐algue brune introduite le long des côtes bretonnes. PhD thesis, Ecologie, Environnement. Sorbonne Université, Paris.

[eva12647-bib-0050] Grulois, D. , Lévêque, L. , & Viard, F. (2011). Mosaic genetic structure and sustainable establishment of the invasive kelp *Undaria pinnatifida* within a bay (Bay of St‐Malo, Brittany). CBM – Cahiers de Biologie Marine, 52(4), 485.

[eva12647-bib-0051] Guillemin, M. L. , Faugeron, S. , Destombe, C. , Viard, F. , Correa, J. A. , & Valero, M. (2008). Genetic variation in wild and cultivated populations of the haploid diploid red alga *Gracilaria chilensis*: How farming practices favor asexual reproduction and heterozygosity. Evolution, 62(6), 1500–1519. 10.1111/j.1558-5646.2008.00373.x 18346220

[eva12647-bib-0052] Houston, R. D. , Davey, J. W. , Bishop, S. C. , Lowe, N. R. , Mota‐Velasco, J. C. , Hamilton, A. , … Blaxter, M. L. (2012). Characterisation of QTL‐linked and genome‐wide restriction site‐associated DNA (RAD) markers in farmed Atlantic salmon. BMC Genomics, 13(1), 244 10.1186/1471-2164-13-244 22702806 PMC3520118

[eva12647-bib-0053] Hudson, J. , Viard, F. , Roby, C. , & Rius, M. (2016). Anthropogenic transport of species across native ranges: Unpredictable genetic and evolutionary consequences. Biology Letters, 12(10), 20160620 10.1098/rsbl.2016.0620 27729485 PMC5095196

[eva12647-bib-0054] James, K. , & Shears, N. T. (2016). Proliferation of the invasive kelp *Undaria pinnatifida* at aquaculture sites promotes spread to coastal reefs. Marine Biology, 163(2), 1–12. 10.1007/s00227-015-2811-9

[eva12647-bib-0055] Jeffery, N. W. , Bradbury, I. R. , Stanley, R. R. E. , Wringe, B. F. , Wyngaarden, M. V. , Lowen, J. B. , … DiBacco, C. (2018). Genome‐wide evidence of environmentally mediated secondary contact of European green crab (*Carcinus maenas*) lineages in eastern North America. Evolutionary Applications, 10.1111/eva.12601 PMC5999199 29928296

[eva12647-bib-0056] Jeffery, N. W. , DiBacco, C. , Wringe, B. F. , Stanley, R. R. E. , Hamilton, L. C. , Ravindran, P. N. , & Bradbury, I. R. (2017). Genomic evidence of hybridization between two independent invasions of European green crab (*Carcinus maenas*) in the Northwest Atlantic. Heredity, 119, 154–165. 10.1038/hdy.2017.22 28422135 PMC5555096

[eva12647-bib-0057] Jombart, T. , & Ahmed, I. (2011). adegenet 1.3‐1: New tools for the analysis of genome‐wide SNP data. Bioinformatics, 27(21), 3070–3071. 10.1093/bioinformatics/btr521 21926124 PMC3198581

[eva12647-bib-0058] Krueger‐Hadfield, S. A. , Kollars, N. M. , Byers, J. E. , Greig, T. W. , Hammann, M. , Murray, D. C. , … Sotka, E. E. (2016). Invasion of novel habitats uncouples haplo‐diplontic life cycles. Molecular Ecology, 25(16), 3801–3816. 10.1111/mec.13718 27286564

[eva12647-bib-0059] Leclerc, J.‐C. , & Viard, F. (2018). Habitat formation prevails over predation in influencing fouling communities. Ecology and Evolution, 8(1), 477–492. 10.1002/ece3.3654 29321887 PMC5756867

[eva12647-bib-0060] Lischer, H. E. , & Excoffier, L. (2011). PGDSpider: An automated data conversion tool for connecting population genetics and genomics programs. Bioinformatics, 28(2), 298–299. 22110245 10.1093/bioinformatics/btr642

[eva12647-bib-0061] Luu, K. , Bazin, E. , & Blum, M. G. (2017). pcadapt: An R package to perform genome scans for selection based on principal component analysis. Molecular Ecology Resources, 17(1), 67–77. 10.1111/1755-0998.12592 27601374

[eva12647-bib-0062] Marchini, A. , Ferrario, J. , & Minchin, D. (2015). Marinas may act as hubs for the spread of the pseudo‐indigenous bryozoan *Amathia verticillata* (Delle Chiaje, 1822) and its associates. Scientia Marina, 79(3), 355–365. 10.3989/scimar.04238.03A

[eva12647-bib-0063] Martin, M. (2011). Cutadapt removes adapter sequences from high‐throughput sequencing reads. EMBnet.journal, 17(1), 10–12. https://doi.org/10.14806/ej.17.1.200

[eva12647-bib-0064] Megina, C. , González‐Duarte, M. M. , López‐González, P. J. , & Piraino, S. (2013). Harbours as marine habitats: Hydroid assemblages on sea‐walls compared with natural habitats. Marine Biology, 160(2), 371–381. 10.1007/s00227-012-2094-3

[eva12647-bib-0065] Minchin, D. (2007a). Aquaculture and transport in a changing environment: Overlap and links in the spread of alien biota. Marine Pollution Bulletin, 55(7), 302–313. https://doi.org/doi: 10.1016/j.marpolbul.2006.11.017 17223137 10.1016/j.marpolbul.2006.11.017

[eva12647-bib-0066] Minchin, D. (2007b). Rapid coastal survey for targeted alien species associated with floating pontoons in Ireland. Aquatic Invasions, 2(1), 63–70. 10.3391/ai

[eva12647-bib-0067] Minchin, D. , & Nunn, J. (2014). The invasive brown alga *Undaria pinnatifida* (Harvey) Suringar, 1873 (Laminariales: Alariaceae), spreads northwards in Europe. BioInvasions Records, 3(2), 57–63. 10.3391/bir

[eva12647-bib-0068] Mineur, F. , Johnson, M. , & Maggs, C. (2008). Macroalgal introductions by hull fouling on recreational vessels: Seaweeds and sailors. Environmental Management, 42(4), 667–676. 10.1007/s00267-008-9185-4 18704562

[eva12647-bib-0069] Molnar, J. L. , Gamboa, R. L. , Revenga, C. , & Spalding, M. D. (2008). Assessing the global threat of invasive species to marine biodiversity. Frontiers in Ecology and the Environment, 6(9), 485–492. 10.1890/070064

[eva12647-bib-0070] Murphy, J. T. , Johnson, M. P. , & Viard, F. (2017). A theoretical examination of environmental effects on the life cycle schedule and range limits of the invasive seaweed *Undaria pinnatifida* . Biological invasions, 19(2), 691–702. 10.1007/s10530-016-1357-1

[eva12647-bib-0071] Nunes, A. L. , Katsanevakis, S. , Zenetos, A. , & Cardoso, A. C. (2014). Gateways to alien invasions in the European seas. Aquatic Invasions, 9(2), 133–144. 10.3391/ai

[eva12647-bib-0072] Nyberg, C. D. , & Wallentinius, I. (2005). Can species traits be used to predict marine macroalgal introductions? Biological Invasions, 7, 265–279. 10.1007/s10530-004-0738-z

[eva12647-bib-0073] Oksanen, J. , Blanchet, F. G. , Kindt, R. , Legendre, P. , Minchin, P. R. , O'hara, R. , … Wagner, H. (2015). vegan: Community ecology package. R package version 2.0‐10. 2013.

[eva12647-bib-0074] Pannell, J. R. , Auld, J. R. , Brandvain, Y. , Burd, M. , Busch, J. W. , Cheptou, P. O. , … Winn, A. A. (2015). The scope of Baker's law. New Phytologist, 208(3), 656–667. 10.1111/nph.13539 26192018

[eva12647-bib-0075] Perez, R. , Lee, J. Y. , & Juge, C. (1981). Observations sur la biologie de l'algue japonaise *Undaria pinnatifida* (Harvey) Suringar introduite accidentellement dans l'Etang de Thau. Science et Pêche, 315, 1–12.

[eva12647-bib-0076] Raj, A. , Stephens, M. , & Pritchard, J. K. (2014). fastSTRUCTURE: Variational inference of population structure in large SNP data sets. Genetics, 197(2), 573–589. 10.1534/genetics.114.164350 24700103 PMC4063916

[eva12647-bib-0101] R Core Team . (2016). R: A language and environment for statistical computing. Vienna, Austria: R Foundation for Statistical Computing Retrieved from http://www.R-project.org/.

[eva12647-bib-0077] Rius, M. , Turon, X. , Bernard, G. , Volckaert, F. , & Viard, F. (2015). Marine invasion genetics: From spatial and temporal patterns to evolutionary outcomes. Biological Invasions, 17(3), 869–885. 10.1007/s10530-014-0792-0

[eva12647-bib-0078] Ros, M. , Vasquez‐Luis, M. , & Guerra‐Gardia, J. (2013). The role of marinas and recreational boating in the occurrence and distribution of exotic caprellids (Crustacea: Amphipoda) in the Western Mediterranean: Mallorca Island as a case study. Journal of Sea Research, 83, 94–103. 10.1016/j.seares.2013.04.004

[eva12647-bib-0079] Rousset, F. (1997). Genetic differentiation and estimation of gene flow from *F*‐statistics under isolation by distance. Genetics, 145(4), 1219–1228. 9093870 10.1093/genetics/145.4.1219 PMC1207888

[eva12647-bib-0080] Rousset, F. (2008). genepop'007: A complete re‐implementation of the genepop software for Windows and Linux. Molecular Ecology Resources, 8(1), 103–106. 10.1111/j.1471-8286.2007.01931.x 21585727

[eva12647-bib-0081] Saarman, N. P. , & Pogson, G. H. (2015). Introgression between invasive and native blue mussels (genus *Mytilus*) in the central California hybrid zone. Molecular Ecology, 24(18), 4723–4738. 10.1111/mec.13340 26230080

[eva12647-bib-0082] Sakai, A. K. , Allendorf, F. W. , Holt, J. S. , Lodge, D. M. , Molofsky, J. , With, K. A. , … Ellstrand, N. C. (2001). The population biology of invasive species. Annual Review of Ecology and Systematics, 32(1), 305–332. 10.1146/annurev.ecolsys.32.081501.114037

[eva12647-bib-0083] Savini, D. , Occhipinti‐Ambrogi, A. , Marchini, A. , Tricarico, E. , Gherardi, F. , Olenin, S. , & Gollasch, S. (2010). The top 27 animal alien species introduced into Europe for aquaculture and related activities. Journal of Applied Ichthyology, 26(s2), 1–7. 10.1111/j.1439-0426.2010.01503.x

[eva12647-bib-0084] Seebens, H. , Blackburn, T. M. , Dyer, E. E. , Genovesi, P. , Hulme, P. E. , Jeschke, J. M. , … Arianoutsou, M. (2017). No saturation in the accumulation of alien species worldwide. Nature Communications, 8, 14435 10.1038/ncomms14435 PMC5316856 28198420

[eva12647-bib-0085] Sliwa, C. , Johnson, C. R. , & Hewitt, C. L. (2006). Mesoscale dispersal of the introduced kelp *Undaria pinnatifida* attached to unstable substrata. Botanica Marina, 49, 396–405.

[eva12647-bib-0086] Sotka, E. E. , Baumgardner, A. W. , Bippus, P. M. , Destombe, C. , Duermit, E. A. , Endo, H. , … Krueger‐Hadfield, S. A. (2018). Combining niche shift and population genetic analyses predicts rapid phenotypic evolution during invasion. Evolutionary Applications, 11, 781–793. 10.1111/eva.12592 29875819 PMC5978718

[eva12647-bib-0087] South, P. M. , Floerl, O. , Forrest, B. M. , & Thomsen, M. S. (2017). A review of three decades of research on the invasive kelp Undaria pinnatifida in Australasia: An assessment of its success, impacts and status as one of the world's worst invaders. Marine Environmental Research, 131, 243–257. 10.1016/j.marenvres.2017.09.015 28958575

[eva12647-bib-0088] South, P. M. , & Thomsen, M. S. (2016). The ecological role of invading *Undaria pinnatifida*: An experimental test of the driver–passenger models. Marine Biology, 163(8), 175 10.1007/s00227-016-2948-1

[eva12647-bib-0089] Stoffel, M. A. , Esser, M. , Kardos, M. , Humble, E. , Nichols, H. , David, P. , & Hoffman, J. I. (2016). inbreedR: An R package for the analysis of inbreeding based on genetic markers. Methods in Ecology and Evolution, 7(11), 1331–1339. 10.1111/2041-210X.12588

[eva12647-bib-0200] Storey, J. (2002). A direct approach to false discovery rates. Journal of the Royal Statistical Society, Series B, 64, 479–498.

[eva12647-bib-0090] Thomsen, M. S. , Wernberg, T. , South, P. M. , & Schiel, D. R. (2016). Non‐native seaweeds drive changes in marine coastal communities around the world In Hu Z.‐M. & Fraser C. (Eds.), Seaweed phylogeography (pp. 147–185). Dordrecht: Springer 10.1007/978-94-017-7534-2

[eva12647-bib-0091] Troost, K. (2010). Causes and effects of a highly successful marine invasion: Case‐study of the introduced Pacific oyster *Crassostrea gigas* in continental NW European estuaries. Journal of Sea Research, 64(3), 145–165. 10.1016/j.seares.2010.02.004

[eva12647-bib-0092] Valentine, J. P. , & Johnson, C. R. (2003). Establishment of the introduced kelp *Undaria pinnatifida* in Tasmania depends on disturbance to native algal assemblages. Journal of Experimental Marine Biology and Ecology, 295, 63–90. 10.1016/S0022-0981(03)00272-7

[eva12647-bib-0093] Valentine, J. P. , Magierowski, R. H. , & Johnson, C. R. (2008). Mechanisms of invasion: Establishment, spread and persistence of introduced seaweed populations In Johnson C.R. (Ed.), Seaweed invasions (pp. 31–40). Berlin: Walter de Gruyter.

[eva12647-bib-0094] Valero, M. , Destombe, C. , Mauger, S. , Ribout, C. , Engel, C. R. , Daguin‐Thiebaut, C. , & Tellier, F. (2011). Using genetic tools for sustainable management of kelps: A literature review and the example of *Laminaria digitata* . CBM – Cahiers de Biologie Marine, 52(4), 467.

[eva12647-bib-0095] Valero, M. , Guillemin, M.‐L. , Destombe, C. , Jacquemin, B. , Gachon, C. M. M. , Badis, Y. , … Faugeron, S. (2017). Perspectives on domestication research for sustainable seaweed aquaculture. Perspectives in Phycology, 4(1), 33–46. 10.1127/pip/2017/0066

[eva12647-bib-0096] Viard, F. , David, P. , & Darling, J. A. (2016). Marine invasions enter the genomic era: Three lessons from the past, and the way forward. Current Zoology, 62(6), 629–642. 10.1093/cz/zow053 29491950 PMC5804250

[eva12647-bib-0097] Voisin, M. , Engel, C. R. , & Viard, F. (2005). Differential shuffling of native genetic diversity across introduced regions in a brown alga: Aquaculture vs. maritime traffic effects. Proceedings of the National Academy of Sciences of the United States of America, 102(15), 5432–5437. 10.1073/pnas.0501754102 15802466 PMC556235

[eva12647-bib-0098] Wallentinus, I. (2007). Alien species alert: Undaria pinnatifida (wakame or japanese kelp) (8774820559). ICES Cooperative Research Report No. 283. 36 pp.

[eva12647-bib-0099] Whitlock, M. C. , & Lotterhos, K. E. (2015). Reliable detection of loci responsible for local adaptation: Inference of a null model through trimming the distribution of F ST. The American Naturalist, 186(S1), S24–S36. 10.1086/682949 26656214

[eva12647-bib-0100] Xing, K. , Gao, M. , & Li, H. (2014). Genetic differentiation between natural and hatchery populations of Manila clam (*Ruditapes philippinarum*) based on microsatellite markers. Genetics and Molecular Research, 13(1), 237–245. 10.4238/2014.January.17.7 24535849

