# Supplementary material for: Population genomics of the introduced and cultivated Pacific kelp Undaria pinnatifida: Marinas—not farms—drive regional connectivity and establishment in natural rocky reefs
Source: Evol Appl. 2018 Jun 14;11(9):1582–97. doi: 10.1111/eva.12647 (PMC6183462; doi:10.1111/eva.12647)

Supplementary Information

Table S1 Samples, genetic diversity and selfing rate computed with 10 microsatellites

The sample label is composed of the habitat type (M=marina, R=natural rocky reef habitat, F= farm (cultivated population)), bay number code and year of sampling, as shown in Figure 1 in the main text. For each locality, the number of study individuals, the expected heterozygosity (H_E), the estimate of fixation index F_{IS}, and the probability of the exact test for Hardy-Weinberg equilibrium are provided. Selfing rates (s) estimated with the g_2 value (David et al. 2007) are also given.

Sample	Habitat	Locality (Bay)	N_{ind}	H_E (SE)	F_{IS}	P_{HW}	s
M1-15	Marina	Port-Navalo (La Trinité)	32	0.311 (0.076)	0.316	0.001	0.591
M2-15	Marina	Port Haliguen (Quiberon)	32	0.343 (0.052)	0.509	<0.001	0.552
M3-15	Marina	Port d'Etel (Etel)	32	0.404 (0.065)	0.257	0.101	0.078
R3-15	Rocky reef	Magouër Nord (Etel)	32	0.407 (0.051)	0.195	0.567	0.154
M4-15	Marina	Port de Loctudy (Loctudy)	32	0.126 (0.051)	0.378	<0.001	0.551
R4-15	Rocky reef	Karreg Saoz (Loctudy)	32	0.108 (0.058)	0.131	0.286	NA
M5-15	Marina	Port Le Guilvinec (Le Guilvinec)	32	0.275 (0.078)	0.185	0.109	0.100
R5-15	Rocky reef	Le Guilvinec Château (Le Guilvinec)	32	0.322 (0.075)	0.137	0.245	0.060
M6-15	Marina	Moulin Blanc (Brest)	32	0.395 (0.060)	0.470	<0.001	0.471
M6-05	Marina	Moulin Blanc (Brest)	40	0.378 (0.069)	0.345	<0.001	0.385
M7-15	Marina	Port Aber Wrac'h (Aber Wrac'h)	32	0.303 (0.079)	0.113	0.152	0.149
R7-15	Rocky reef	Breach ver (Aber Wrac'h)	32	0.284 (0.072)	0.156	0.162	0.046
M8-15	Marina	Port Bloscon (Morlaix)	32	0.471 (0.063)	0.210	0.004	0.350
F8-15	Farm	Ferme Biocean (Morlaix)	32	0.245 (0.082)	-0.058	0.107	0.055
F8-09	Farm	Ferme Biocean (Morlaix)	16	0.231 (0.083)	-0.054	0.614	0.150
F8-05	Farm	Ferme Biocean (Morlaix)	26	0.170 (0.069)	0.028	0.856	0.627
Ra8-15	Rocky reef	Guerhéon (Morlaix)	28	0.363 (0.079)	0.063	0.070	0.053
Rb8-15	Rocky reef	Men Guen (Morlaix)	30	0.108 (0.064)	0.198	0.145	NA
M9-15	Marina	Port Trieux (Bréhat)	27	0.432 (0.066)	0.160	0.077	0.301
R9-15	Rocky reef	Chenal Ile (Bréhat)	32	0.248 (0.072)	0.174	0.999	0.206
M10-15	Marina	Port St. Quay (St. Quay Portrieux)	32	0.195 (0.064)	0.327	<0.001	0.361
R10-15	Rocky reef	Ile Harbour (St. Quay Portrieux)	32	0.151 (0.065)	0.257	0.098	0.576
M11-15	Marina	Port St. Cast (St. Cast le Guildo)	32	0.395 (0.069)	0.328	<0.001	0.348
R11-15	Rocky reef	Roche de l'Etendrée (Frehel)	32	0.324 (0.067)	-0.040	0.516	0.237
M12-15	Marina	Port Bas Sablons (St. Malo)	32	0.460 (0.058)	0.349	<0.001	0.247
M12-09	Marina	Port Bas Sablons (St. Malo)	23	0.410 (0.063)	0.087	0.517	0.049
M12-05	Marina	Port Bas Sablons (St. Malo)	30	0.425 (0.063)	0.195	<0.001	0.256
F12-15	Farm	C-weed (St. Malo)	32	0.234 (0.076)	0.076	0.478	0.197
F12-09	Farm	C-weed (St. Malo)	29	0.256 (0.070)	0.042	<0.001	0.477
F12-05	Farm	C-weed (St. Malo)	23	0.353 (0.077)	-0.060	0.731	0.132
Ra12-15	Rocky reef	Fort National (St. Malo)	32	0.384 (0.060)	0.057	0.239	0.032
Ra12-09	Rocky reef	Fort National (St. Malo)	30	0.388 (0.055)	0.233	<0.001	0.087
Ra12-05	Rocky reef	Fort National (St. Malo)	43	0.371 (0.065)	0.217	<0.001	0.154
Rb12-15	Rocky reef	Le Grand Murier (St. Malo)	32	0.439 (0.054)	0.103	0.040	0.196
Rb12-09	Rocky reef	Le Grand Murier (St. Malo)	32	0.388 (0.061)	0.048	0.203	0.113
Rb12-05	Rocky reef	Le Grand Murier (St. Malo)	30	0.434 (0.061)	0.038	0.201	0.178

Table S2. Pairwise F_{ST} (below diagonal) computed between samples collected in 2015, A) with RAD-seq panel, B) with microsatellites. All values were significant at the 5% level except those indicated in bold. P-values were adjusted for multiple comparisons to control for the false discovery rate (FDR).

A)

	**M1-15**	**M2-15**	**M3-15**	**R3-15**	**M4-15**	**R4-15**	**M5-15**	**R5-15**	**M6-15**	**M7-15**	**R7-15**	**M8-15**	**F8-15**	**Ra8-15**	**Rb8-15**	**M9-15**	**R9-15**	**M10-15**	**R10-15**	**M11-15**	**R11-15**	**M12-15**	**F12-15**	**Ra12-15**	**Rb12-15**
**M1-15**	0.000																								
**M2-15**	0.207	0.000																							
**M3-15**	0.268	0.277	0.000																						
**R3-15**	0.259	0.278	**-0.003**	0.000																					
**M4-15**	0.372	0.379	0.353	0.321	0.000																				
**R4-15**	0.434	0.446	0.420	0.416	**-0.001**	0.000																			
**M5-15**	0.279	0.288	0.310	0.315	0.378	0.451	0.000																		
**R5-15**	0.276	0.292	0.299	0.292	0.435	0.465	0.098	0.000																	
**M6-15**	0.234	0.226	0.254	0.258	0.359	0.434	0.268	0.267	0.000																
**M7-15**	0.263	0.266	0.287	0.284	0.376	0.437	0.102	0.116	0.235	0.000															
**R7-15**	0.272	0.276	0.299	0.299	0.389	0.448	0.118	0.129	0.254	0.071	0.000														
**M8-15**	0.148	0.150	0.183	0.201	0.251	0.358	0.185	0.160	0.127	0.149	0.158	0.000													
**F8-15**	0.412	0.405	0.435	0.417	0.550	0.565	0.429	0.453	0.406	0.413	0.415	0.301	0.000												
**Ra8-15**	0.250	0.246	0.277	0.284	0.352	0.433	0.286	0.281	0.250	0.258	0.261	0.157	0.068	0.000											
**Rb8-15**	0.537	0.539	0.541	0.509	0.698	0.653	0.439	0.500	0.514	0.442	0.459	0.391	0.668	0.505	0.000										
**M9-15**	0.178	0.199	0.204	0.205	0.325	0.381	0.124	0.146	0.177	0.084	0.097	0.075	0.367	0.190	0.445	0.000									
**R9-15**	0.296	0.299	0.297	0.285	0.425	0.452	0.263	0.298	0.282	0.240	0.258	0.188	0.468	0.292	0.574	0.187	0.000								
**M10-15**	0.465	0.461	0.490	0.451	0.623	0.614	0.487	0.516	0.454	0.480	0.488	0.356	0.617	0.451	0.727	0.421	0.522	0.000							
**R10-15**	0.366	0.370	0.395	0.354	0.506	0.519	0.302	0.312	0.358	0.275	0.295	0.252	0.531	0.356	0.608	0.235	0.379	0.421	0.000						
**M11-15**	0.261	0.234	0.263	0.239	0.495	0.478	0.302	0.333	0.233	0.269	0.292	0.126	0.484	0.242	0.708	0.199	0.347	0.571	0.438	0.000					
**R11-15**	0.224	0.225	0.248	0.243	0.359	0.426	0.277	0.269	0.207	0.247	0.258	0.135	0.398	0.237	0.516	0.170	0.280	0.439	0.345	0.216	0.000				
**M12-15**	0.108	0.124	0.142	0.165	0.205	0.326	0.167	0.136	0.110	0.128	0.139	0.044	0.271	0.134	0.379	0.045	0.155	0.315	0.218	0.076	0.094	0.000			
**F12-15**	0.414	0.407	0.436	0.418	0.551	0.567	0.433	0.456	0.408	0.417	0.419	0.303	0.017	0.071	0.671	0.367	0.471	0.619	0.532	0.486	0.401	0.273	0.000		
**Ra12-15**	0.148	0.161	0.183	0.185	0.256	0.354	0.209	0.186	0.150	0.169	0.182	0.073	0.311	0.161	0.438	0.086	0.188	0.370	0.275	0.125	0.134	0.033	0.313	0.000	
**Rb12-15**	0.126	0.134	0.168	0.160	0.242	0.337	0.178	0.157	0.119	0.146	0.152	0.042	0.300	0.140	0.425	0.056	0.173	0.359	0.262	0.105	0.107	**-0.002**	0.302	0.040	0.000

B)

	**M1-15**	**M2-15**	**M3-15**	**R3-15**	**M4-15**	**R4-15**	**M5-15**	**R5-15**	**M6-15**	**M7-15**	**R7-15**	**M8-15**	**F8-15**	**Ra8-15**	**Rb8-15**	**M9-15**	**R9-15**	**M10-15**	**R10-15**	**M11-15**	**R11-15**	**M12-15**	**F12-15**	**Ra12-15**	**Rb12-15**
**M1-15**	0.000																								
**M2-15**	0.195	0.000																							
**M3-15**	0.207	0.141	0.000																						
**R3-15**	0.213	0.169	**0.015**	0.000																					
**M4-15**	0.373	0.379	0.395	0.402	0.000																				
**R4-15**	0.305	0.314	0.380	0.395	0.167	0.000																			
**M5-15**	0.258	0.145	0.231	0.221	0.455	0.436	0.000																		
**R5-15**	0.261	0.169	0.258	0.269	0.444	0.394	0.127	0.000																	
**M6-15**	0.211	0.153	0.089	0.127	0.417	0.383	0.185	0.154	0.000																
**M7-15**	0.241	0.158	0.214	0.226	0.427	0.370	0.149	0.094	0.110	0.000															
**R7-15**	0.371	0.276	0.344	0.345	0.527	0.492	0.261	0.128	0.233	0.080	0.000														
**M8-15**	0.255	0.177	0.151	0.160	0.423	0.408	0.206	0.196	0.142	0.143	0.178	0.000													
**F8-15**	0.305	0.284	0.275	0.290	0.475	0.452	0.331	0.299	0.231	0.240	0.320	0.247	0.000												
**Ra8-15**	0.192	0.130	0.161	0.177	0.347	0.302	0.187	0.155	0.127	0.131	0.224	0.150	0.046	0.000											
**Rb8-15**	0.493	0.382	0.402	0.405	0.640	0.573	0.495	0.444	0.419	0.439	0.476	0.372	0.611	0.472	0.000										
**M9-15**	0.208	0.131	0.151	0.143	0.394	0.380	0.099	0.073	0.078	0.086	0.125	0.086	0.194	0.089	0.397	0.000									
**R9-15**	0.217	0.118	0.213	0.255	0.480	0.424	0.213	0.257	0.195	0.241	0.396	0.220	0.384	0.229	0.534	0.226	0.000								
**M10-15**	0.237	0.127	0.259	0.263	0.431	0.365	0.143	0.224	0.234	0.247	0.389	0.284	0.329	0.165	0.557	0.202	0.169	0.000							
**R10-15**	0.202	0.161	0.282	0.295	0.359	0.183	0.261	0.288	0.274	0.256	0.411	0.320	0.345	0.184	0.507	0.263	0.254	0.128	0.000						
**M11-15**	0.143	0.137	0.103	0.146	0.340	0.289	0.182	0.126	0.063	0.101	0.219	0.120	0.181	0.071	0.390	0.081	0.190	0.224	0.204	0.000					
**R11-15**	0.250	0.218	0.159	0.160	0.449	0.422	0.212	0.201	0.142	0.107	0.204	0.121	0.340	0.224	0.372	0.109	0.299	0.359	0.337	0.099	0.000				
**M12-15**	0.122	0.108	0.077	0.073	0.293	0.265	0.123	0.123	0.068	0.115	0.208	0.110	0.182	0.063	0.349	0.040	0.198	0.154	0.165	0.044	0.115	0.000			
**F12-15**	0.349	0.300	0.288	0.310	0.519	0.501	0.372	0.347	0.252	0.303	0.378	0.274	**0.008**	0.075	0.636	0.231	0.406	0.353	0.391	0.225	0.396	0.218	0.000		
**Ra12-15**	0.150	0.160	0.126	0.119	0.341	0.307	0.119	0.097	0.090	0.081	0.187	0.144	0.191	0.080	0.395	0.053	0.232	0.201	0.209	0.039	0.088	0.028	0.248	0.000	
**Rb12-15**	0.148	0.099	0.062	0.063	0.338	0.320	0.102	0.110	0.058	0.085	0.190	0.097	0.199	0.082	0.384	0.027	0.185	0.175	0.209	0.043	0.076	**0.006**	0.240	0.026	0.000

Table S3. Hierarchical analysis of the molecular variance (AMOVA) performed on the RAD-seq panel to examine the spatial (habitat type) and temporal effects on genetic structure.

Significant values of the fixation indices are indicated in bold. For the temporal analysis of the marinas, there is only a single entry for 2009 because sample M6 was not sampled in 2009.

Hierarchical design	% Variance	*F*-Statistics	*P*-value
**Habitat**			
Samples from 2015, 3 groups (3 groups: marinas, farms, natural sites)			
Among habitats	4.61	Fct = 0.046	**<0.001**
Among populations within habitat	27.89	Fsc = 0.292	**<0.001**
Among individuals within populations	12.06	Fis = 0.179	**<0.001**
Within individuals	55.44	Fit = 0.446	**<0.001**
Samples from 2015, 2 groups (2 groups: marinas and natural sites)			
Among habitats	-1.14	Fct = -0.011	1.000
Among populations within habitat	29.86	Fsc = 0.295	**<0.001**
Among individuals within populations	14.06	Fis = 0.197	**<0.001**
Within individuals	57.22	Fit = 0.428	**<0.001**
**Temporal**			
Natural sites (2 localities) grouped by year (3 groups: 2005, 2009, 2015)			
Among years	-1.34	Fct = -0.013	1.000
Among populations within year	4.96	Fsc = 0.049	**<0.001**
Among individuals within populations	12.46	Fis = 0.129	**<0.001**
Within individuals	83.92	Fit = 0.161	**<0.001**
Marinas (2 localities) grouped by year (3 groups: 2005, 2009, 2015)			
Among groups	-6.17	Fct = -0.062	1.000
Among populations within groups	14.08	Fsc = 0.133	**<0.001**
Among individuals within populations	25.79	Fis = 0.280	**<0.001**
Within individuals	66.30	Fit = 0.337	**<0.001**
Farms (2 localities), grouped by year (3 groups: 2005, 2009, 2015)			
Among years	27.04	Fct = 0.270	**<0.001**
Among populations within year	12.79	Fsc = 0.175	**<0.001**
Among individuals within populations	-0.89	Fis = -0.015	1.000
Within individuals	61.05	Fit = 0.389	**<0.001**

Figure S1. Clustering analyses on the RAD-seq panel, using the *snmf* function of *LEA* (Frichot and Francois 2015), over A) the 2015 dataset, and B) the Bay of St. Malo scale (temporal analysis).

Each individual is represented by a vertical line divided into K coloured segments, the length of which indicates the individual's membership fraction to each of K clusters. Individuals are grouped according to their sampling locality (ordered along a south to north gradient) for the regional scale analysis, and according to locality and year of sampling for the analysis at the bay scale. Locality codes correspond to those specified in Table 1. Note that conversely to fastSTRUCTURE (see figures in the main text), the *snmf* algorithm does not rely on Hardy-Weinberg equilibrium assumptions, and is particularly appropriate to use with inbred species (Frichot, Mathieu, Trouillon, Bouchard, & Francois (2014). Fast and efficient estimation of individual ancestry coefficients. *Genetics, 196*(4), 973-983).

A) Regional scale - 2015

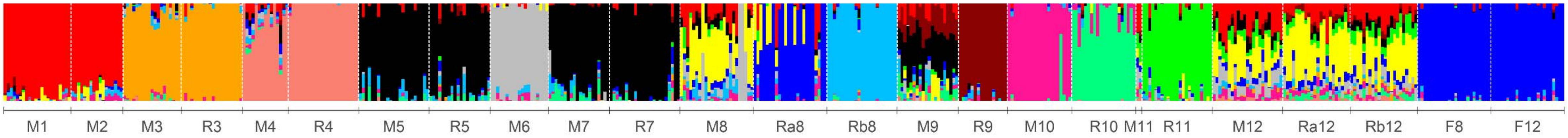

B) Bay scale (St-Malo, no. 12) – Temporal analysis

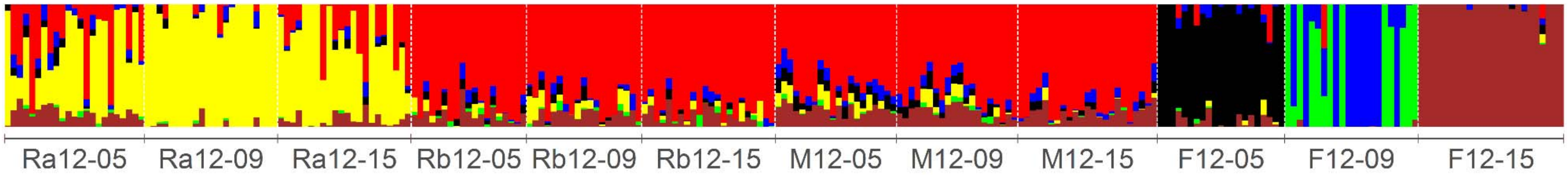

Figure S2. Discriminant analyses of principal components for the A) RAD-seq panel (10,615 loci) and B) microsatellite panel (10 loci)

The first two discriminant functions are displayed in the top plot and the second and third functions are displayed in the bottom plot. The sample codes, shown in the legend in the bottom right corner of each panel, correspond to those given in Table 1, and the symbols (shape and colour) refer to those displayed in Fig. 1.

A) RAD-seq panel

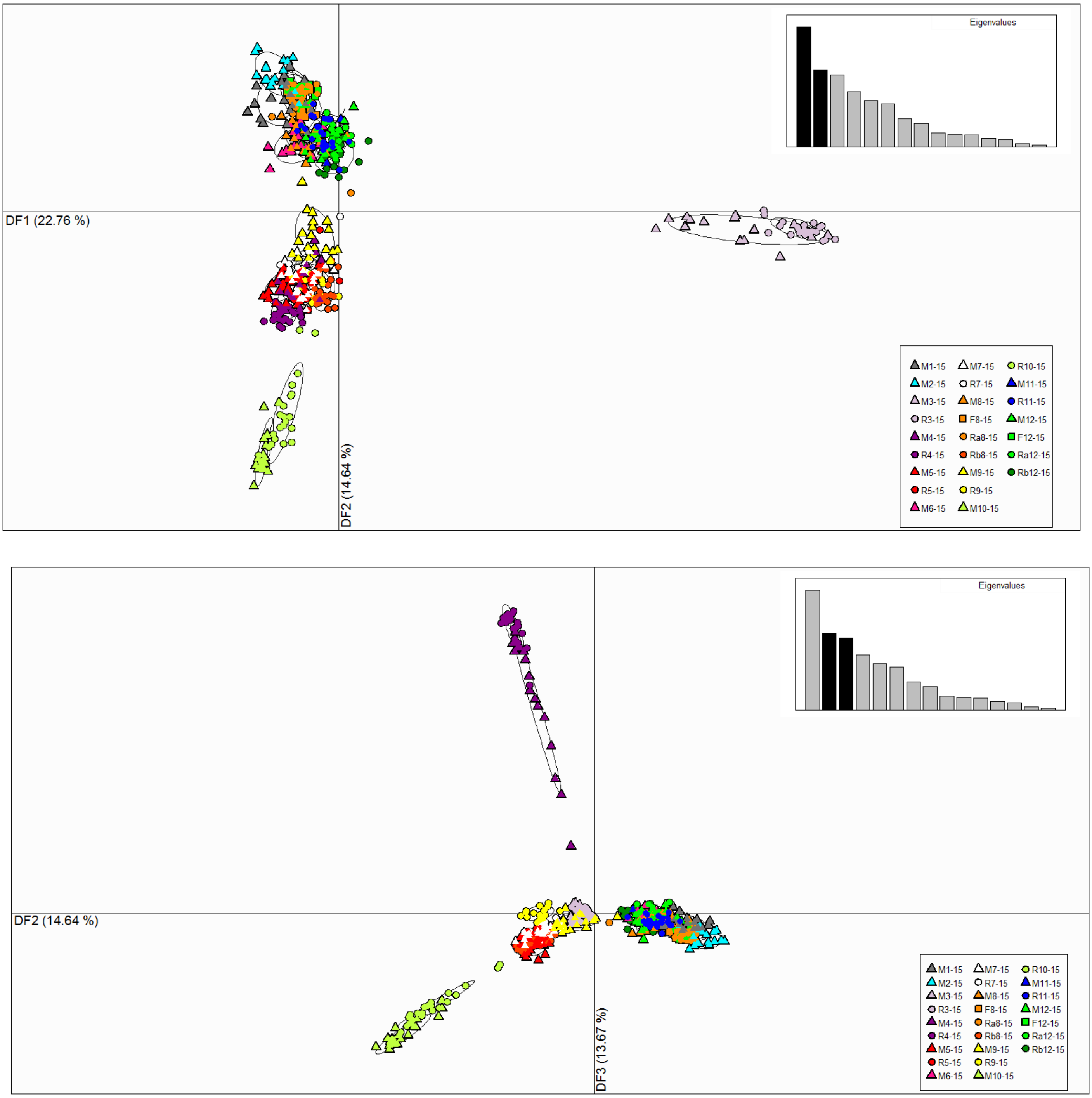

B) Microsatellite panel

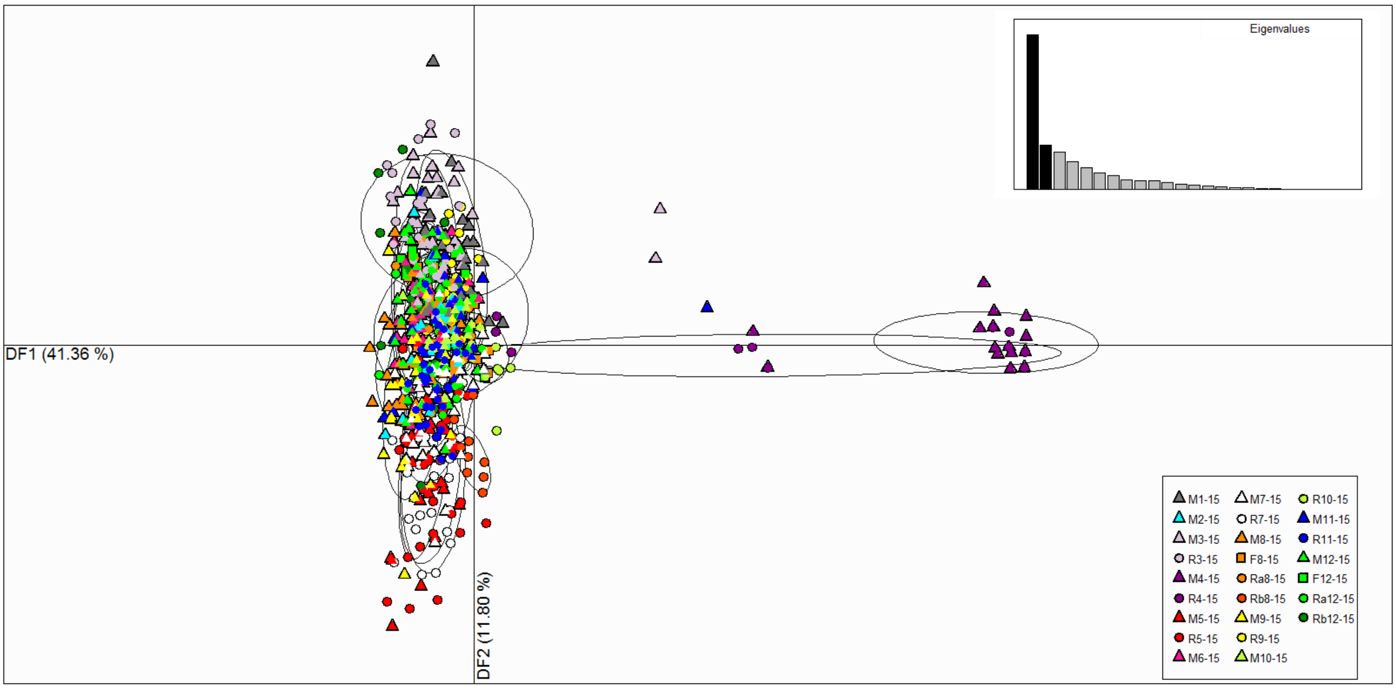

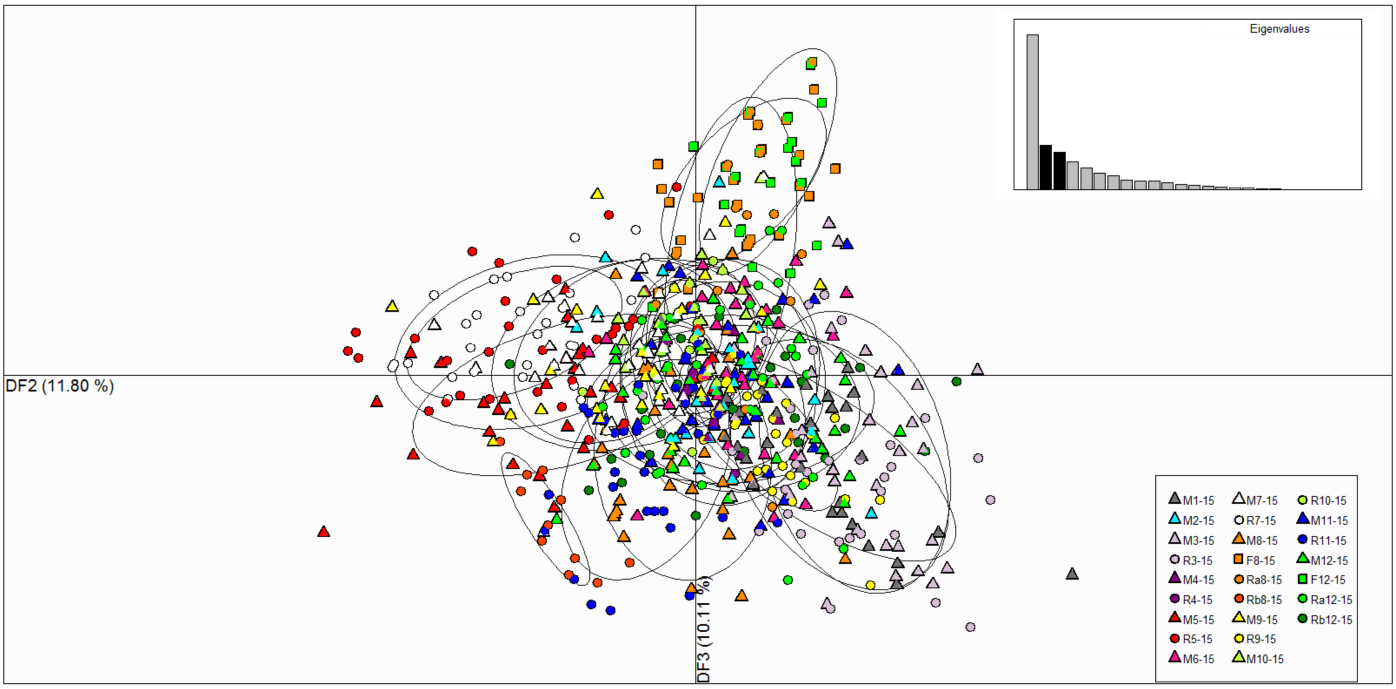

Figure S3. Bayesian clustering analyses made for the microsatellite panel over the whole dataset sampled in 2015 (A and B), and at the level of the Bay of St. Malo (bay no. 12 in Fig. 1, C and D) using STRUCTURE (A and C) or INSTRUCT (B and D).

Each individual is represented by a vertical line divided into K coloured segments, the length of which indicates the individual's membership fraction to each of K clusters. Individuals are grouped according to their sampling locality (ordered along a south to north gradient) for the regional scale analysis, and according to locality and year of sampling for the analysis at the bay scale. Locality codes correspond to those specified in Table 1. STRUCTURE relies on Hardy-Weinberg equilibrium assumptions, unlikely to be met in a selfing species. Conversely, INSTRUCT does not rely on this assumption and jointly estimates ancestry coefficient and selfing rates.

A) Microsatellite panel - Regional scale 2015- STRUCTURE

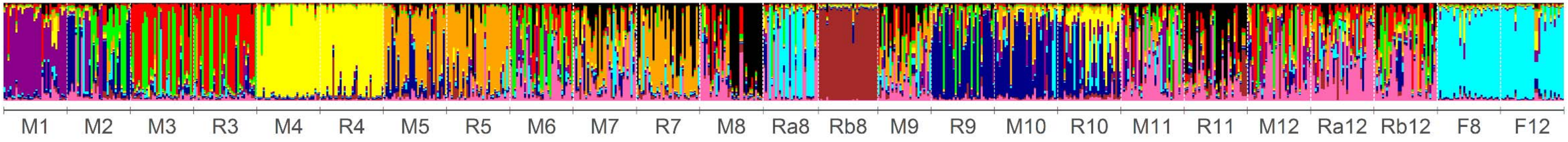

B) Microsatellite panel - Regional scale 2015– INSTRUCT

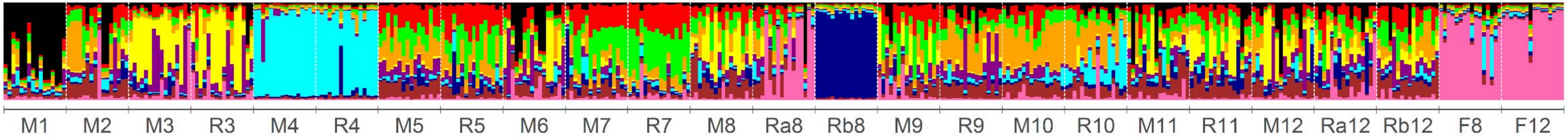

C) Microsatellite panel – Bay scale (St. Malo), temporal analysis – STRUCTURE

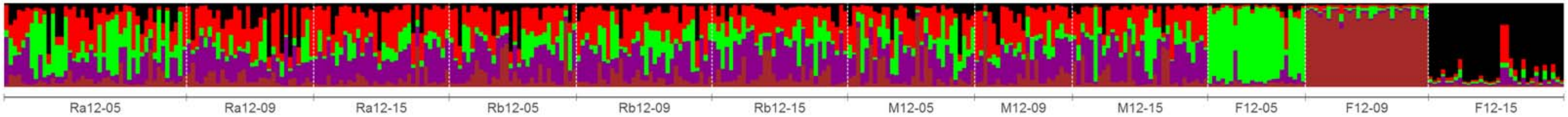

D) Microsatellite panel – Bay scale (St. Malo), temporal analysis - INSTRUCT

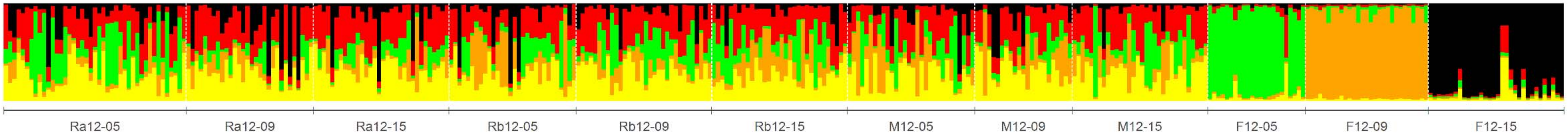

Figure S4. Venn diagrams showing the number of positive selection outliers detected using five different methods

A) Sample set 1 (marina and rocky reef samples collected in 2015), and B) sample set 2 (samples collected in 2015 in bays no. 8 and 12), with the RAD-seq panel.

Note that OutFLANK is not included on the diagrams because no single outlier was detected at the 5% q-value threshold.

A) Sample set 1

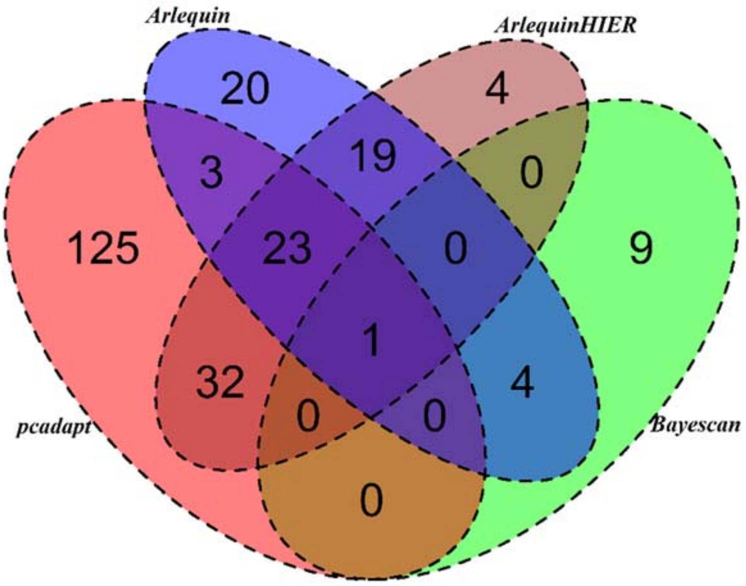

B) Sample set 2

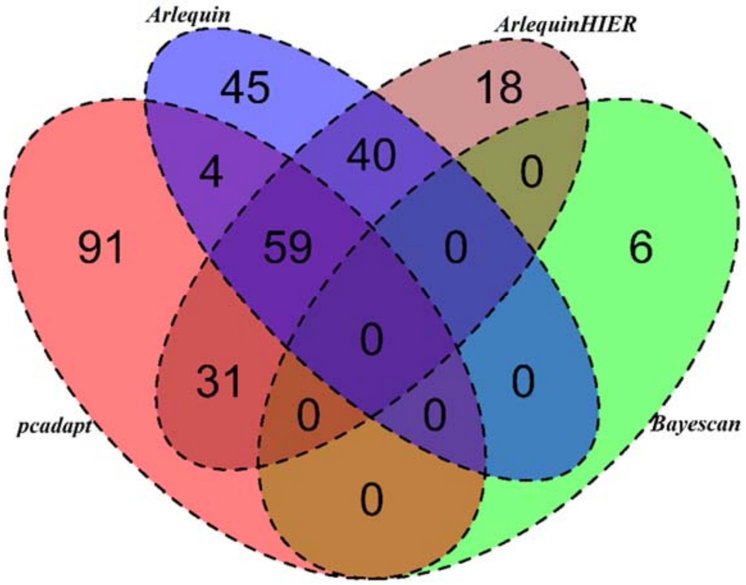

Figure S5. Bayesian clustering analyses with the fastStructure software using alternative RAD-seq dataset

RAD-seq panel with sample set 1 (marinas and natural sites sampled in 2015) used for outlier detection, with analyses done using A) 64 loci identified as positive selection outliers by OutFLANK with a q-value of 0.056 (see text), and B) the putative "neutral" loci, i.e. all SNP loci except the 240 outlier loci detected by at least one of the five methods used (including OutFLANK at the 5.6% threshold). OutFLANK is the most conservative outlier detection method.

Each individual is represented by a vertical line divided into K coloured segments, the length of which indicates the individual's membership fraction to each of K clusters. Individuals are grouped according to their sampling locality (ordered along a south to north gradient).

A) Outlier SNPs panel – Sample set 1 (2015; marinas and natural rocky habitats)

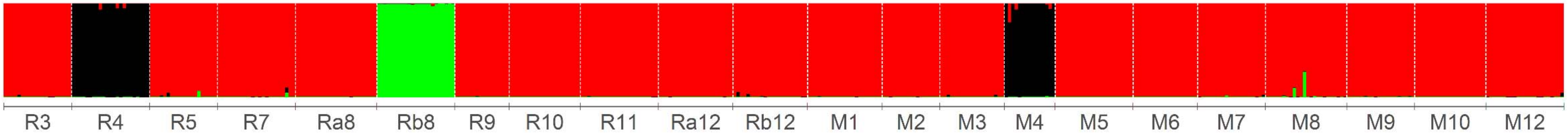

B) "Neutral" SNPs panel – Sample set 1 (2015; marinas and natural rocky habitats)

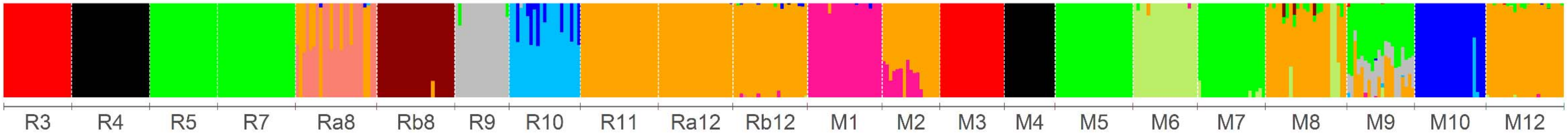

Supplement: Supplementary file 1 [file EVA-11-1582-s001.pdf]
